# Barriers to cancer treatment for people experiencing socioeconomic disadvantage in high-income countries: a scoping review

**DOI:** 10.1186/s12913-024-11129-2

**Published:** 2024-05-28

**Authors:** Amber Bourgeois, Tara Horrill, Ashley Mollison, Eleah Stringer, Leah K. Lambert, Kelli Stajduhar

**Affiliations:** 1 Institute for Aging & Lifelong Health, University of Victoria, PO Box 1700, Victoria, BC V8V 2Y2 Canada; 2 BC Cancer, Nursing and Allied Health Research and Knowledge Translation, 686 West Broadway, Vancouver, BC V5Z 1G1 Canada; 3 College of Nursing, University of Manitoba, 89 Curry Place, Winnipeg, MB R3T 2N2 Canada; 4 School of Nursing, University of British Columbia, 2211 Wesbrook Mall T201, Vancouver, BC V6T 2B5 Canada

**Keywords:** Health inequities, Access, Cancer treatment, Vulnerable populations, Social disadvantage

## Abstract

**Background:**

Despite advances in cancer research and treatment, the burden of cancer is not evenly distributed. People experiencing socioeconomic disadvantage have higher rates of cancer, later stage at diagnoses, and are dying of cancers that are preventable and screen-detectable. However, less is known about barriers to accessing cancer treatment.

**Methods:**

We conducted a scoping review of studies examining barriers to accessing cancer treatment for populations experiencing socioeconomic disadvantage in high-income countries, searched across four biomedical databases. Studies published in English between 2008 and 2021 in high-income countries, as defined by the World Bank, and reporting on barriers to cancer treatment were included.

**Results:**

A total of 20 studies were identified. Most (*n* = 16) reported data from the United States, and the remaining included publications were from Canada (*n* = 1), Ireland (*n* = 1), United Kingdom (*n* = 1), and a scoping review (*n* = 1). The majority of studies (*n* = 9) focused on barriers to breast cancer treatment. The most common barriers included: inadequate insurance and financial constraints (*n* = 16); unstable housing (*n* = 5); geographical distribution of services and transportation challenges (*n* = 4); limited resources for social care needs (*n* = 7); communication challenges (*n* = 9); system disintegration (*n* = 5); implicit bias (*n* = 4); advanced diagnosis and comorbidities (*n* = 8); psychosocial dimensions and contexts (*n* = 6); and limited social support networks (*n* = 3). The compounding effect of multiple barriers exacerbated poor access to cancer treatment, with relevance across many social locations.

**Conclusion:**

This review highlights barriers to cancer treatment across multiple levels, and underscores the importance of identifying patients at risk for socioeconomic disadvantage to improve access to treatment and cancer outcomes. Findings provide an understanding of barriers that can inform future, equity-oriented policy, practice, and service innovation.

**Supplementary Information:**

The online version contains supplementary material available at 10.1186/s12913-024-11129-2.

## Background

Cancer is a major public health concern, accounting for nearly 10 million deaths worldwide in 2020 [1, 2]. Globally, cancer incidence and mortality is steadily increasing; yet, the cancer burden is not evenly distributed [1, 2]. Inequities in cancer outcomes have been made increasingly visible by the COVD-19 pandemic in high-income countries, particularly among people and populations disproportionately impacted by multiple barriers to accessing healthcare. However, evidence has long shown alarming differences in cancer outcomes between population groups stratified along a socioeconomic gradient: the lower the socioeconomic position, the worse one’s health [3–5]. For example, people whose lived/living experience is shaped by socioeconomic disadvantage (i.e., lower-income, poverty, homelessness) have higher incidence rates of certain cancers, are more likely to be diagnosed at advanced-stages, and are more likely to die from cancers that are preventable and treatable [6–10]. 

Timely access to cancer care across the continuum (e.g., prevention, screening, diagnosis, treatment, survivorship, end-of-life) is important in reducing the cancer burden [4, 9–20]. However, the broader social, economic, political, and historical contexts intersect in ways that often pushes people who experience socioeconomic disadvantage to the margins, resulting in poorer access to cancer care services [9–21, 22]. Research on addressing barriers to cancer care services has largely focused on barriers to cancer screening [14–17, 18]. Unmet social determinants of health and competing priorities of daily living (e.g., poverty, housing instability, food insecurity, transportation difficulties, material deprivation), and poor access to primary healthcare services are among some of the conditions that result in lower screening uptake [9, 14–17, 20]. Additionally, health literacy- a strong predictor of health status- is lower among people who experience socioeconomic disadvantage [24, 25]. In cancer care, patients are required to understand complex information, consent to health interventions, seek help in a timely manner, and is considered to be a prerequisite for shared and informed decision making. Consequently, lower health literacy may contribute to a limited awareness of the importance of cancer prevention and screening, resulting in worse outcomes [24, 25]. In part, the solution to enhancing equitable access to cancer care is by addressing the barriers to prevention and screening, however; not all cancers are screen-detectable.^26^ Additionally, patients who face socioeconomic disadvantage may have managed to get through the layers of inequities at the screening and diagnosis phase, and then can still go on to face more barriers at the treatment stage [27]. Access to treatment is particularly salient in reducing cancer morbidity and mortality as inequitable access to cancer treatment has led to widening differences in survival and reduced quality of life [12, 13, 19, 20, 23, 27, 28–32]. While many of these barriers may indeed be the same, a nuanced understanding of the particular contexts at the treatment stage is also required to inform policy and practice innovation.

In this review, we have conceptualized cancer treatment as approved active and/or maintenance cancer treatments including drugs or procedures used to cure, reduce, or stop the progression of cancer (see Table 1) [32–35]. Inequitable access to cancer treatment refers to whether people receive treatment, and also encompasses factors that decrease treatment efficacy such as incomplete courses of treatment, missed doses, treatment delays, or nonadherence to treatment. These are all known to increase the risk of tumor progression or recurrence, lead to poor outcomes, and potentially contribute to the inefficient use of healthcare resources [28, 29]. A growing body of research suggests that people who face socioeconomic disadvantage are less likely to receive treatment [12, 14–16, 27, 28–37], and are also more likely to experience greater delays in starting treatment [12, 13, 38]. Additionally, they are also less likely to receive high-quality treatment and often receive treatment outside of recommended guidelines [15, 39], are under-represented in clinical trial enrollment [40], have higher rates of non-adherence, and discontinue treatment earlier than recommended [10–15, 18, 20, 27–30, 41, 42]. While these findings highlight the association between disparities in treatment access and survival, knowledge of the specific barriers leading to these outcomes remains somewhat limited as no singular study has comprehensively explored the full spectrum of contributing factors.

### Aims

The aim of this scoping review was to summarize and map peer-reviewed literature on barriers to accessing cancer treatment for people who experience socioeconomic disadvantage in high-income countries. Our objective was to understand common barriers to cancer treatment that are relevant to inform policy, innovate cancer treatment services, and identify areas for future research. This review included a multidisciplinary team composed of researchers with internationally-recognized expertise in health equity, cancer research, and healthcare providers (HCPs) in the cancer care sector.

### Theoretical perspectives

Our review is theoretically informed by critical social justice and health equity perspectives, which recognize access to healthcare as a fundamental human right [5, 42–47]. The concepts of critical social justice foreground how we have come to understand health equity, and bring into focus how differences in health are produced and sustained by social arrangements (e.g., institutions, relationships) and structures that are embedded within socio-cultural, political, economic, and historical contexts [5, 43, 44, 46, 47]. We understand health inequities as those avoidable and possibly remediable differences in health outcomes that are shaped by unjust social arrangements and structures [5, 42–45]. 

## Study design and methods

In recognition of this complex topic, we employed scoping review methodology rather than a systematic review to map and summarize the existing evidence on barriers to cancer treatment for people who experience socioeconomic disadvantage [47–50]. Additionally, advancing equitable access to cancer treatment is still emerging, and first we must understand how the literature conceptualizes barriers to cancer treatment so that we can identify gaps in the knowledge [50]. As such, a scoping review was deemed the best approach. The methods used in this review are based on six iterative stages, as outlined in the work of Arksey & O’Malley [40] and Levac and colleagues [49], and are expanded below. We have reported our process using the Preferred Reporting Items for Systematic Reviews and Meta-Analyses extension for scoping reviews (PRISMA-ScR) guidelines [51]. 

### Stage 1: identification of the research question

In the first stage, we identified the concepts under study and target population to answer the research question: *what are the barriers to cancer treatment for populations experiencing socioeconomic disadvantage with a cancer diagnosis in high income-countries?* Theoretically informed by social justice and health equity discourse, we were particularly interested in (but not limited to) barriers to cancer treatment stemming from social and structural determinants of health. Key concepts within our review included access to healthcare, cancer treatment, barriers, and socioeconomic disadvantage (Table 1).

**Table 1:** Definition of key concepts

Concept	Definition
Access to healthcare	Access to healthcare is a key determinant of health and has been conceptualized as the interplay between the delivery, availability, and distribution of healthcare services and the ability of an individual or group to obtain care (e.g., physically access care, perceive the need for care, overcome socioeconomic barriers in accessing care) to achieve the best health outcomes [52, 53].
Cancer treatment	Due to advancements in research, technology, and precision medicine, cancer treatment has become increasingly complex. During our search, we limited our review to approved active and/or maintenance cancer treatments including drugs or procedures used to cure, reduce, or stop the progression of cancer. These included: surgery, radiation, chemotherapy, targeted therapy, immunotherapy, stem cell or bone marrow transplant, and hormone therapy [32–35].
Barriers to cancer treatment	We conceptualized barriers as modifiable determinants that contribute to a disparity in accessing or adhering to cancer treatment, which in turn result in worse cancer outcomes [54, 55].
Socioeconomic disadvantage	Although there is no universally accepted definition, socioeconomic disadvantage refers to people and/or population groups who are living in less favorable social and economic circumstances compared to the majority in the same society. Features of socioeconomic disadvantage may include economic disparity (e.g., lower levels of income, poverty, homelessness), and lower educational attainment and occupational standing. People experiencing socioeconomic disadvantage are often exposed to poor environmental conditions (e.g., crowded or inadequate housing), more frequently experience adverse social conditions (e.g., racism, stigma, gender discrimination), and adverse early childhood experiences [56, 57].

### Stage 2: identification of relevant Studies

A comprehensive search strategy that identified key words and subject headings was co-developed by the study team and two biomedical librarians with expertise in the cancer care sector. Search strategies were peer-reviewed by third librarian using the Peer Review of Electronic Search Strategies (PRESS) checklist. We searched four biomedical databases (Ovid Medline (R); Ovid Embase; Ovid EBM Reviews-Cochrane Database of Systematic Reviews; and EBSCO CINAHL) during the months of September through November 2021. The search filters were limited to English language resources published in 2008 onward, reflecting a marked shift in attention to health equity following the landmark report published by World Health Organizations (WHO) Commission of the Social Determinants of Health [5]. To limit the search to include high-income countries [58], a filter was adapted from the NICE OECD countries’ geographic search filter for Ovid Medline [59]. The principal author conducted backward searching in bibliographies of systematic reviews and scoping reviews identified in our search, which did not result in additional references. Details of the search terms are available in Supplementary File 1.

### Stage 3: study selection

**Fig. 1:**
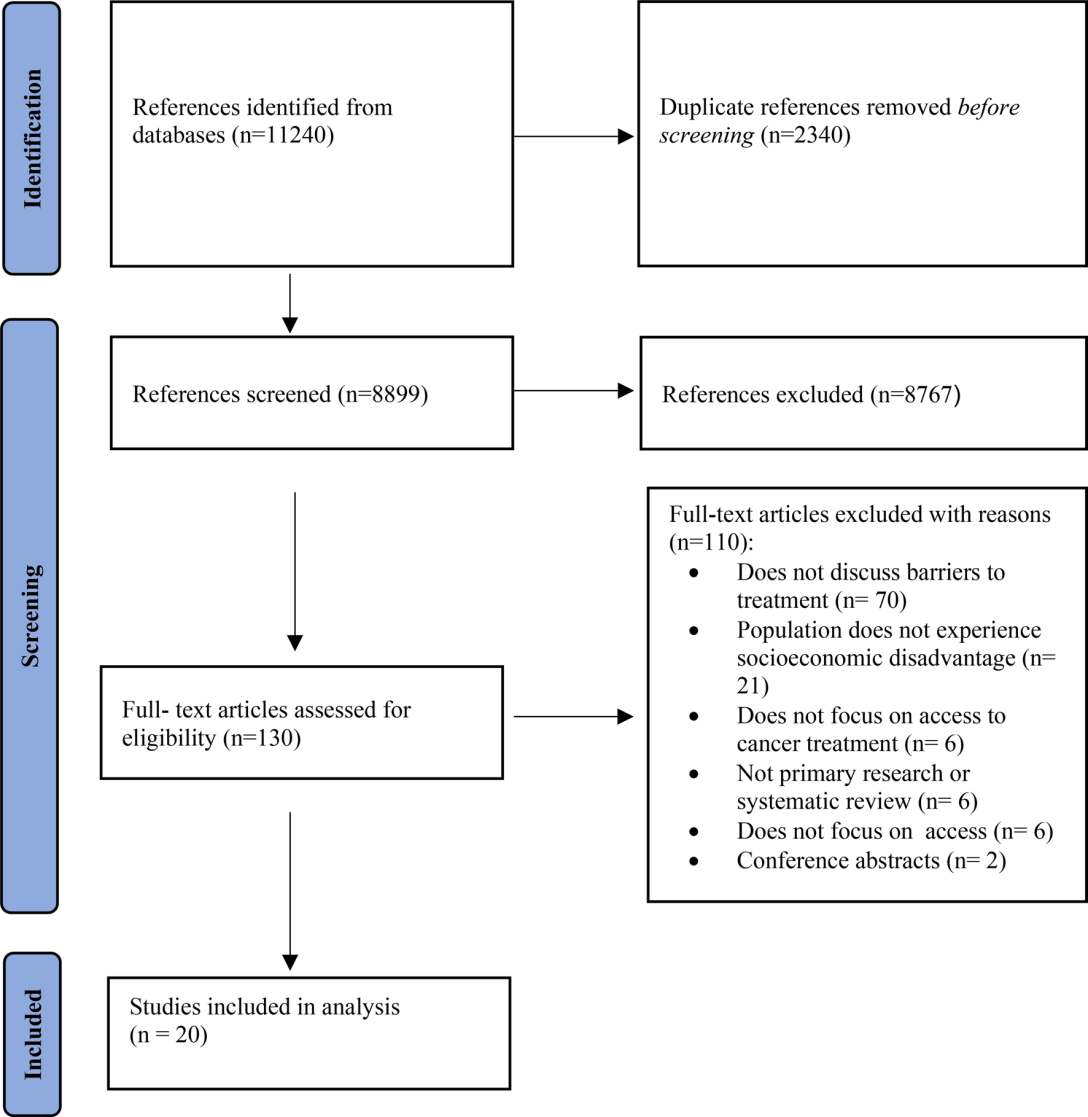
PRISMA Flow diagram for study selection

After removal of duplicate articles and title screening for sources not relevant to our study population, we conducted a two-stage screening process. First, abstracts were reviewed by two reviewers, who applied the inclusion/exclusion criteria (Table 2). Second, full text articles meeting all inclusion criteria were reviewed by two reviewers. The study team met weekly during the study selection process to discuss and clarify decisions during the full text screening stage. Conflicts were resolved through third party discussion with the principal investigator if a consensus could not be reached. We used Covidence software (www.covidence.org) to organize the study selection process, reported in Fig. 1 according to the PRISMA extension for scoping reviews [51]. 

**Table 2:** Inclusion criteria

Criterion		Inclusion	Exclusion
Language		English primary or translated into English.	All other
Date		2008–2021	All other
Country		Research takes place in a high-income country as defined by the World Bank (2020).	All other
Publication type		Peer-reviewed primary research publications or literature reviews (i.e., systematic reviews or scoping reviews).	Case reports, discussion papers, commentaries, thesis/dissertations, conference proceedings, slide presentations, news stories.
Population	Description	Population focus is persons experiencing socioeconomic disadvantage with a cancer diagnosis.	Population of focus is not experiencing socioeconomic disadvantage and/or does not have a cancer diagnosis.
	Age range	Research participants are adults ≥ 18 years of age.	Research participants < 18 years of age.
Publication		Publication includes target population AND has a substantive focus on access and/or adherence to active and/or maintenance cancer treatment (i.e., as discussed in the research findings).	Research does not have a substantive focus or make mention of barriers to accessing and/or adherence to cancer treatment: • Barriers to treatment are an incidental finding or were not a primary focus or direct outcome of the study. • Barriers to treatment are only mentioned in the background or discussion, but not discussed in the research findings. • Barriers to treatment are not explicitly named (i.e., we cannot interpret).
Cancer care sector		Focuses on barriers to receiving or adhering to active and/or maintenance cancer treatment.	Focus is external to the cancer sector or outside active and/or maintenance cancer treatment (i.e., prevention, screening, diagnosis, supportive care, complementary or alternative medicine, surveillance, end-of-life care).

### Stage 4: charting (extracting the data)

Data extraction of included articles was completed in Covidence by one reviewer (AB) using an extraction form developed iteratively by the study team. We extracted data for the following domains: publication characteristics (i.e., title, author(s), year, country where the study was conducted), study aims, methodology, sample size, setting, population description, cancer characteristics (e.g., cancer subtype, treatment type), study results on barriers to accessing cancer treatment. Additionally, root causes of barriers to cancer treatment and future recommendations were extracted if they were explicitly identified in the article. A secondary reviewer (TH) verified a portion (*n* = 5, 25%) of the articles for quality.

### Stage 5: collating, summarizing, and reporting the results

In the final stage, we analyzed extracted data to map barriers in accessing cancer treatment, reported the results of our review as a narrative summary, and determined implications of the review findings [47–50]. We begin by describing characteristics of the included studies, followed by our thematic analysis of barriers to cancer treatment among people experiencing socioeconomic disadvantage. Finally, we discuss the implications of findings and gaps in the current research landscape.

## Results

*Characteristics of the included articles.* A total of 20 studies were included in our scoping review (Supplementary File 2). 80% of the studies originated from the United States. Notably, there were very few studies published from countries with universal access to healthcare (Table 3). The study settings varied considerably. The majority of studies involved a single recruitment setting from people living with cancer. Data sources were derived from safety net hospitals, cancer treatment facilities, hospital inpatient units, cancer registry data, state funded medical assistance program, non-government homeless health services program, and surveys. In terms of examining barriers to treatment, breast cancer was the most commonly studied tumor type, followed by studies involving multiple tumor sites (Table 4). Cancer treatment is personalized to the individual and often multimodal, which was also reflected in the published research. None of the studies explored barriers to receiving immunotherapy, targeted therapy, and stem cell or bone marrow transplant, or access to clinical trials (Table 5).

**Table 3:** Summary of study characteristics (*n* = 20)

Study characteristics		Author/year	Number of publications
Study methodology	Qualitative	Borraya et al., 2020; Bowen et al., 2013; Byrne et al., 2017; Darby et al., 2009; Gould et al., 2009; Jerome et al., 2021; Lineback et al., 2017; Leal et al., 2018; Oduro et al., 2012; Smith et al., 2014	10 (50%)
	Quantitative	Crawford et al., 2009; Costas-Muniz et al., 2016;Emerson, et al., 2020; Facer et al., 2020; Levitz et al., 2015; Liu et al., 2013; Tarazi et al., 2017	7 (35%)
	Mixed methods	Festa et al., 2019; Noel et al., 2015	2 (10%)
	Scoping review	Lawrie et al., 2020	1 (5%)
Publication year	2008–2012	Crawford et al., 2009; Darby et al., 2009; Gould et al., 2009; Oduro et al., 2012	4 (20%)
	2013–2016	Bowen et al., 2013; Costas-Muniz et al., 2016; Levitz et al., 2015; Liu et al., 2013; Noel et al., 2015; Smith et al., 2014	6 (30%)
	2017–2021	Borraya et al., 2020; Byrne et al., 2017; Emerson, et al., 2020; Facer et al., 2020; Festa et al., 2019; Jerome et al., 2021; Lawrie et al., 2020; Leal et al., 2018; Lineback et al., 2017; Tarazi et al., 2017	10 (50%)
Country	United States	Borraya et al., 2020; Bowen et al., 2013; Costas-Muniz et al., 2016; Darby et al., 2009; Emerson, et al., 2020; Facer et al., 2020; Festa et al., 2019; Jerome et al., 2021; Leal et al., 2018; Levitz et al., 2015; Lineback et al., 2017; Liu et al., 2013; Noel et al., 2015; Oduro et al., 2012; Smith et al., 2014; Tarazi et al., 2017	16 (80%)
	Canada	Gould et al., 2009	1 (5%)
	United Kingdom	Crawford et al., 2009	1 (5%)
	Ireland	Byrne et al., 2017	1 (5%)
	Not applicable	Lawrie et al., 2020^a^	1 (5%)
Number of recruitment settings	Single	Borraya et al., 2020; Bowen et al., 2013;Byrne et al., 2017; Crawford et al., 2009;Emerson et al., 2020; Facer et al., 2020; Festa et al., 2019; Jerome et al., 2021; Leal et al., 2018; Levitz et al., 2015; Liu et al., 2013 Oduro et al., 2012; Smith et al., 2014; Tarazi et al., 2017	14 (70%)
	Multiple	Costas- Muniz et al., 2016; Darby et al., 2009; Lineback et al., 2017; Noel et al., 2015	4 (20%)
	Not specified	Gould et al., 2009	1 (5%)
	Not applicable	Lawrie et al., 2020^a^	1 (5%)
Sample size	< 25	Bowen et al., 2013 (*n* = 16); Byrne et al., 2017 (*n* = 17); Festa et al., 2019 (*n* = 24); Jerome et al., 2021 (*n* = 20); Smith et al., 2014 (*n* = 15)	5 (25%)
	25- <100	Borrayo et al., 2020 (*n* = 29); Darby et al., 2009 (*n* = 36); Facer et al., 2020 (*n* = 32); Gould et al., 2009 (*n* = 41); Leal et al., 2018 (*n* = 30); Lineback et al., 2017 (*n* = 88) Oduro et al., 2012 (*n* = 60)	7(35%)
	100 - < 5000	Costas-Muniz et al., 2016 (*n* = 1098); Emerson et al., 2020 (*n* = 2841); Levitz et al., 2015 (*n* = 2319); Liu et al., 2013 (*n* = 921); Noel et al., 2015 (*n* = 885)	5 (25%)
	≥ 5000	Crawford et al., 2009 (*n* = 34,923); Tarazi et al., 2017 (*n* = 19,100)	2 (10%)
	Not applicable	Lawrie et al., 2020^a^	1 (5%)
Study participants	People living with cancer	Bowen et al., 2013; Byrne et al., 2017; Costas-Muniz et al., 2016; Crawford et al., 2009; Darby et al., 2009; Emerson et al., 2020; Facer et al., 2020; Festa et al., 2019; Gould et al., 2009; Jerome et al., 2021; Leal et al., 2018; Levitz et al., 2015; Lineback et al., 2017; Liu et al., 2013; Oduro et al., 2012; Tarazi et al., 2017	16 (80%)
	Cancer care providers (e.g., social workers and financial care counselors)	Smith et al., 2014	1 (5%)
	Multiple	Borrayo et al., 2020; Noel et al., 2015	2 (10%)
	Not applicable	Lawrie et al., 2020^a^	1 (5%)

a. One study was a scoping review

**Table 4:** Summary of study cancer characteristics (*n* = 20)

Cancer characteristic		Author/year	Number of publications
Cancer subtype	Breast	Bowen et al., 2013; Bryne et al., 2017; Darby et al., 2009; Emerson et al., 2020; Festa et al., 2020; Jerome et al., 2021; Liu et al., 2013; Noel et al., 2015; Tarazi et al., 2017	9 (45%)
	Colorectal	Leal et al., 2018; Levitz et al., 2015	2 (10%)
	Lung	Crawford et al., 2009	1 (5%)
	Esophageal	Lineback et al., 2017	1 (5%)
	Prostate	Oduro et al., 2012	1 (5%)
	Multiple	Borrayo et al., 2020^a^; Costas-Muniz et al., 2016^b^; Facer et al., 2020^c^; Gould et al., 2009^d^	4 (20%)
	Not specified	Smith et al., 2014	1 (5%)
	Not applicable	Lawrie et al., 2020^e^	1 (5%)
Treatment modality	Radiation therapy	Facer et al., 2020	1 (5%)
	Chemotherapy	Levitz et al., 2015	1 (5%)
	Hormone therapy	Liu et al., 2013	1 (5%)
	Multi-modal	Borrayo et al., 2020^f^; Bowne et al., 2013^g^; Costas-Muniz et al., 2016^h^; Crawford et al., 2009^i^; Emerson et al., 2020^i^; Lineback et al., 2017^i^; Noel et al., 2015^i^; Festa et al., 2020^j^; Tarazi et al., 2017^j^; Gould et al., 2009^k^; Oduro et al., 2012^k^	11 (55%)
	Not specified	Byrne et al., 2017; Darby et al., 2009; Jerome et al., 2021; Lawrie et al., 2020; Leal et al., 2018; Smith et al., 2014	6 (30%)
	Not applicable	Lawrie et al., 2020^e^	1 (5%)

a. One study reported on lung cancer and head and neck cancer(s)

b. One study reported on breast, gastrointestinal, prostate, gynecological cancer(s)

c. One study reported on lung, head-and-neck, gastrointestinal, unknown, hematology, prostate, breast, cervical, central nervous system, and skin cancer(s)

d. One study reported on breast and gynecological cancer(s)

e. One study was a scoping review

f. One study reported on radiation therapy and treatment not specified

g. One study reported on breast cancer surgery (mastectomy vs lumpectomy) and treatment not specified

h. One study reported on radiation and/or chemotherapy

i. Four studies reported on surgery, radiation, and chemotherapy

j. Two studies reported on surgery and chemotherapy

k. Two studies reported on surgery, chemotherapy, radiation, and hormone treatment

### Barriers to accessing cancer treatment

Barriers to accessing healthcare are multidimensional and one established way of analyzing complexity is by distinguishing between micro-meso-macro level considerations [60]. *Macro-*level barriers refer to the upstream social and structural determinants of health such as the political, economic, historical and social conditions that systematically create and sustain inequities. These factors create obstacles to achieving a healthy life and produce barriers to accessing healthcare [60, 61]. Meso-level barriers as those challenges at the point-of-care arising from the way in which cancer care is designed, structured and delivered at the healthcare organizational/institutional level [61, 62]. Lastly, micro-level barriers are the downstream consequences of structural inequities at the macro-level (i.e., poverty, racism, unstable housing) that shape an individual’s social location, interpersonal relationships, and physical health [60, 61, 63]. While the categories in this framework are presented distinctly, they interact and intersect with one another across the spectrum of a person’s cancer experience (Tables 5 and 6).

**Table 5:** Summary of barriers (*n* = 20)

Level	Barrier	Author/year	Number of publications
Macro	Inadequate insurance coverage and financial constraints	Borrayo et al., 2020; Bowen et al. 2013; Byrne et al., 2018; Darby et al., 2009; Emerson et al., 2020; Facer et al., 2021; Gould et al., 2009; Jerome-D’Emilia et al., 2020; Lawrie et al., 2020; Leal et al., 2018; Levitz et al., 2015; Lineback et al., 2017; Liu et al., 2012; Oduro et al., 2012; Smith et al., 2014; Tarazi et al., 2017	16 (80%)
	Unstable housing	Borrayo et al., 2020; Costas-Muniz et al., 2016; Facer et al., 2021; Festa et al., 2020; Lawrie et al., 2020;	5 (25%)
	Geographical distribution of services and transportation challenges	Bowen et al., 2013; Crawford et al., 2009; Emerson et al., 2020; Facer et al., 2021	4 (20%)
	Compounding barriers and their intersections	Costas-Muniz et al., 2016; Emerson et al., 2020; Levitz et al., 2015; Oduro et al., 2012	4 (20%)
Meso	Communication challenges	Borrayo et al., 2020; Bowen et al. 2013; Byrne et al., 2018; Lawrie et al., 2020; Leal et al., 2018; Lineback et al., 2017; Liu et al., 2012; Noel et al., 2015; Oduro et al., 2012	9 (45%)
	Limited resources for social care needs	Borrayo et al., 2020; Byrne et al., 2018; Gould et al., 2009; Lineback et al., 2017; Liu et al., 2012; Oduro et al., 2012; Smith et al., 2014	7 (35%)
	System disintegration	Borrayo et al., 2020; Lawrie et al., 2020; Lineback et al., 2017; Noel et al., 2015; Oduro et al., 2012	5 (25%)
	Implicit bias	Facer et al., 202; Gould et al., 2009; Lawrie et al., 2020; Noel et al., 2015	4 (20%)
Micro	Advanced diagnosis and comorbidities	Borrayo et al., 2020; Bowen et al., 2013; Byrne et al., 2018; Facer et al., 2021; Festa et al., 2020; Lawrie et al., 2020; Liu et al., 2012; Jerome-D’Emilia et al., 2020	8 (40%)
	Psychosocial dimensions and contexts	Borrayo et al., 2020; Bowen et al., 2013; Byrne et al., 2018; Gould et al., 2009;Jerome-D’Emilia et al., 2020	5 (25%)
	Limited social support networks	Borrayo et al., 2020; Costas-Muniz et al., 2016; Levitz et al., 2015	3 (15%)

**Table 6:** Outcome of barriers to treatment (*n* = 20)

Outcome	Author/year	Number of publications
Treatment non-adherence (e.g., missed treatment appointments or skipped doses for hormone therapy)	Costas- Muniz et al., 2016; Darby et al., 2009; Facer et al., 2020; Lawrie et al., 2020; Liu et al., 2013; Smith et al., 2014	6 (30%)
Did not receive treatment	Crawford et al., 2009; Levitz et al., 2015; Lineback et al., 2017; Noel et al., 2015)	4 (20%)
Delay in starting treatment	Jerome et al., 2021; Oduro et al., 2012; Tarazi et al., 2017	3 (15%)
Did not receive treatment that was comparable to their more financially stable counterparts	Bowen et al., 2013	1 (5%)
Multiple treatment outcomes	Borrayo et al., 2020^a^; Byrne et al., 2017^b^; Emerson et al., 2020^c^; Festa et al., 2020^d^; Gould et al., 2009^e^; Leal et al., 2015^f^	6 (30%)

a. One study reported on missed treatment appointments, non- receipt radiation, poor adherence or non-completion

b. One study reported on delay in starting treatment, poor adherence to hormone therapy

c. One study reported on delay in starting treatment and prolonged treatment duration

d. One study reported on delay to first treatment, missed treatment appointments

e. One study reported on not having timely access to first treatment, decided against receiving treatment

f. Delays in starting treatment, non- receipt of treatment

#### Macro-level barriers

Regardless of the country of origin, barriers at the macro-level related to the compounding effects of socioeconomic disadvantage shaped the conditions in which people could access cancer treatment and meet the basic determinants of daily life. Through examining macro-contexts, structural forces such as political and social systems were revealed to create disparities in access to care.

### Inadequate insurance and financial constraints

Insurance coverage for cancer treatment was largely driven by the conditions set forth by political environments, and was identified as a fundamental barrier to accessing care [64]. Within the context of the mixed healthcare (i.e., public and private) system in the United States, for example, inadequate or no insurance coverage to cover medical costs resulted in poor access to treatment [65], poor adherence [66], greater delays in receiving treatment [38, 64, 67, 68], having to receive substandard treatment options based on cost, [69, 70] or foregoing treatment altogether [37]. Many people who experience socioeconomic disadvantage are also faced with precarious working environments. Two studies identified that some people lost their jobs when they were diagnosed with cancer and this meant they also lost their employer-paid health benefits resulting in no longer having insurance to cover their medical costs, which then became a significant barrier to receiving cancer treatment [68, 70]. Likewise, some people with lower levels of income did not meet the income threshold for public health insurance (i.e., Medicaid) and therefore were required to cover the cost of their treatments. The high cost of cancer treatment meant individuals who were already financial strained fell deeper into poverty, often having to choose between receiving treatment or meeting their basic necessitates of daily living [65, 68, 71]. To illustrate, Smith and colleagues [72] found patients who could not afford their oral cancer treatment were cutting their pills in half, thus compromising the efficacy of this treatment. In other cases, patients with lower levels of income and without insurance lost their homes and were forced to live in their automobiles to pay for cancer treatment [72]. 

Yet, even for those who have public medical coverage, people who experienced socioeconomic disadvantage found it more challenging to afford out-of-pocket expenses or hidden costs directly required for their treatment such as supportive care drugs, nutrition supplements, access to allied health resources, childcare to attend appointments, transportation, and travel expenses (i.e., food, lodging, gas, parking) [68–71,73−75]. Consequently, not having these necessary supports led to higher rates of non-adherence [74, 75] and/or meant that systemic therapy was poorly tolerated [73]. 

#### Unstable housing

Safe and adequate housing is a fundamental basic necessity, yet unstable housing was identified as a significant barrier to cancer treatment in one-quarter of the extracted studies (*n* = 5) [65,76–79]. In some cases, loss of employment after a cancer diagnosis made it difficult to afford basic necessitates, resulting in housing instability or threat of eviction [74, 76, 79]. For people living in temporary or transitional housing (e.g., homeless shelters), adherence to systemic therapy and/or radiation was particularly arduous [78]. Inadequate storage available for medications, a threat of theft of belongings, and poor living conditions were often considered incompatible to safely manage the side effects of systemic therapy or recovery from surgery [65, 78, 79]. Over-crowded living quarters in homeless shelters placed unstably-housed and immunocompromised individuals undergoing chemotherapy at a greater risk for serious infection [65]. Consequently, these conditions meant that treatment was often delayed or not offered at all until safe and stable housing could be secured [79]. Moreover, delays in treatment tended to vary along a gradient according to the degree to which individuals experienced housing instability. For example, Festa and colleagues [79] found that women with breast cancer who were chronically homeless (i.e., unhoused for one or more years) experienced greater delays in starting treatment at a safety net hospital for breast cancer compared to those who were either transitionally (i.e., emergency shelter for weeks to months) or episodically (i.e., homeless for less than one year) housed. Although access to safe and stable housing was identified as a pre-requisite for cancer treatment, programs addressing housing needs for cancer patients facing housing instability are nearly nonexistent [76]. A few studies recommended providing temporary housing during treatment as a potential path forward to enabling equitable access to cancer treatment for those who are unstably housed [65, 76]. However, this would require a commitment from multiple levels of government and collaboration between the healthcare and social care system (i.e., housing sector) [65, 76]. 

#### Geographical distribution of services and transportation challenges

Inequities arising from rural/urban differences were cited as a barrier in 20% of the studies (*n* = 4) [36, 38, 69, 77]. In particular, dedicated cancer treatment facilities are often centralized to urban hubs and located within more affluent geographic areas [36]. This distribution of services disproportionately impacts people who experience socioeconomic disadvantage with cancer from rural or remote areas where the additional out-of-pocket expenses associated with travel and transportation challenges have led to missed appointments, treatment delays, or not being able to receive treatment [36, 69]. In particular, radiation is typically administered in a dedicated cancer treatment facility and requires multiple successive visits over the course of days to several weeks. As such, transportation issues were a significant predictor for poor adherence or skipped/missed visits which prolonged the duration of treatment among people who experience socioeconomic disadvantage especially if radiation was part of the treatment plan [38]. 

### Meso-level barriers

Nearly half of the included studies (*n* = 9) cited barriers that originated at the cancer care organizational level [65, 66, 68, 71–74, 77, 78]. Treatment is often less accessible for people who have greater health and social care needs in part because of how cancer treatment is organized, designed, and delivered. To equitably meet the requirements for successful adherence people experiencing socioeconomic disadvantage often require tailored approaches to cancer care; however, inflexible policies, resource constraints, and a ‘fractured’ healthcare system means that overcoming barriers are more challenging [65, 71, 78, 80]. 

#### Limited resources for social care needs

In high-income countries, the cancer care system often has supportive care resources (e.g., nutrition, patient and family counselling, psychiatry, social work, speech-language pathology, physiotherapy) to assist patients undergoing treatment. However, three studies cited there is a scarcity of resources and lack expertise to support patients who experience socioeconomic disadvantage and whose social care needs may also go unnoticed by HCPs [70, 72, 73]. For example, Lineback and colleagues [70] found that although patients with less favorable socioeconomic circumstances with esophageal cancer were provided with a care team such as a case manager or social worker, the cancer hospital itself was less able to solve multiple issues arising from mistrust, poor communication, and financial strain. In particular, in the United States limited resources to assist with financial support (i.e., social work support, stringent eligibility criteria for financial aid programs, poor quality insurance coverage, and less visible clinical space) often led to compromises in the availability of treatment options, or choice of cancer treatment facility [70, 72]. Additionally, people who experience socioeconomic disadvantage may be reluctant to bring up their financial challenges or may be unaware of supportive care services (e.g., coverage for out-of-pocket expenses, psychosocial care, or transportation) available to them leading to higher rates of treatment nonadherence [65, 72, 74]. 

Financial aid to cover the cost of treatment has been widely acknowledged as a key strategy to reduce barriers to receiving cancer treatment, yet, this is only one piece of a complex puzzle. Cancer care programs which focus on improving access beyond insurance coverage are more likely to dismantle barriers at the systems level [68]. For example, providing coverage for other out-of-pocket expenses associated with treatment such as a lunch voucher, parking ticket, or transportation are small gestures with a potentially large impact on accessing treatment [72]. Adherence to systemic therapy regimens may be impacted when people cannot afford their supportive care medications [66]. In other cases, additional resources are often required to get people out of ‘crisis mode’ such as access to lodging. Conducting a social care needs assessment upfront may help to identify these needs early and throughout the treatment continuum [72]. 

#### Communication challenges

Accessing cancer care requires a high degree of self-management, described as ‘self-efficacy’ by two studies, in order to understand written and verbal communication related to cancer treatment and care [66, 78]. The quality of communication and shared understanding of treatment-related decisions between patient and cancer care providers was at the crux of the success or failure of treatment [65]. A breakdown in patient-provider communication was a key barrier to cancer treatment for people facing socioeconomic disadvantage in nearly half of the included studies (*n* = 9), [65, 66, 68–71, 74, 78, 80, 9] compounding issues of poor access particularly for those with lower levels of health literacy, [65, 69, 71, 74, 78, 80] and for those who do not speak the commonly used language [66, 68, 71]. In many cases, a breakdown in communication occurred when cancer care providers used inaccessible medical language, did not take time to answer questions, or build health literacy in their patients [68, 70, 78]. Inaccessible medical language for patients with lower levels of health literacy greatly impacted a patient’s ability to understand key information related to their cancer diagnosis and treatment plan such as what to expect during treatment and how to manage potential side effects [65, 67, 68–71, 74, 78, 80]. In one example, Noel and colleagues [80] identified a disconnection between what was recorded in the electronic medical record (EMR) and the patient’s recount of their treatment options. In this study, one physician recorded in the EMR radiation and chemotherapy were recommended, whereas the patient stated she declined chemotherapy and was unable to relay the details of her consultation [80]. In some cases, poor patient-provider communication led to a reluctance to ask questions or seek a secondary opinion and a greater reliance on outside support such as family members to help manage their care [74]. Additionally, a lack of language-specific written materials was identified as a key issue for many minority groups who did not speak the common language [65, 68, 71, 80]. Many studies (25%) cited that these mismatches in communication often created a feeling of mistrust, leaving patients disempowered and reluctant to access the healthcare system. The net effect of poor communication resulted in treatment delays, higher rates of non-adherence, or the perception from HCPs of patients being less engaged in their treatment plan [67, 69, 70, 74, 78].

#### System disintegration

Lack of care continuity (e.g., multiple and changing HCPs) and fragmentation between cancer care services (e.g., surgery, radiation, systemic therapy, and supportive care) presents patients with challenges navigating what is often referred to as a ‘fractured’ or ‘siloed’ cancer care system. Yet, these challenges are much greater for people who face socioeconomic disadvantage, and who may also experience lower levels of health literacy, language barriers, mental health challenges, or who lack a social support network [60, 65, 78, 80]. For example, Borraya and colleagues [65] indicated ‘underserved’ Hispanic patients with head and neck cancer who faced language barriers and lower levels of health literacy were often unsure of where to go for appointments, why certain tests were required, and what medical resources were available. This led to frustration with many aspects of their care and resulted in missed appointments or not being able to adhere to treatment. Notably, if systems navigation is too difficult, then actions such as keeping clinical appointments or attending medical procedures, will seem less important than other competing priorities and people are less likely to adhere to their treatment regimen [60, 65, 78, 80]. Nevertheless, under the guise of ‘self-efficacy’, has been misinterpreted, framing a person’s ability to navigate the cancer care system as an individual problem, rather than recognizing the cancer systems’ responsibility to ensure equitable access and foster self-efficacy [78]. To address barriers in this category, patient-navigation services have demonstrated some success in streamlining cancer care between treatment modalities (e.g., between surgery, systemic therapy, radiation) [65, 68, 78, 80]. However, patient navigation services are not made equally available between specialties, nor are they consistently taken up across all practice settings. This signals a gap in navigation services, which is often related to limited availability of these resources [80]. 

#### Implicit bias

Lastly, we draw attention to the often less visible and unequal relations of power arising from implicit assumptions and biases in practice, which are reinforced by often inflexible organizational structures and policies. Facer et al. [77] , highlight that evidence-based clinical guidelines do not exist for clinicians who specifically care for people experiencing the multiplicative socioeconomic disadvantage such as people who face homelessness, experience mental health and/or substance use challenges, or lack the social supports or infrastructure deemed necessary for treatment. The lack of evidence-based guidelines requires oncology care providers to make subjective decisions based past experiences. This situation creates opportunities for implicit bias to influence their decision-making regarding a patient’s capacity or living circumstances deemed necessary to make adherence possible. In some cases, people are not deemed eligible for treatment [77, 78, 80]. Higher rates of non-adherence to treatment is more common among people with cancer who experience socioeconomic disadvantage and also lack material resources [77, 78, 80]. Two studies identified patients experiencing homelessness labelled as ‘noncompliant’ when material deprivation such as lack of transportation and access to a phone or computer caused them to miss appointments [77, 78]. Stigma towards people experiencing homelessness is further perpetuated when the reason for these missed appointments went undocumented in the EMR and were not followed up on [77, 78]. Contrary to these assumptions, Lawrie and colleagues [78] found that people experiencing homelessness are quite motivated to persist with the requirements of treatment if they are well supported and aware of the benefits of receiving treatment. Not only are the micro-aggressions produced by implicit bias and stigma harmful and yield poor outcomes, they also silo experiences of care, and further cement feelings of mistrust towards the cancer care system [73, 77, 78]. Gould et al., underline the *“importance that access to health services is not merely instrumental, but also symbolic. When persons who occupy disadvantaged social locations do not receive the same services or resources as others, it is not the absence of the service or response that matters, it is the message, intended or not, that they lack value, that when cancer happens to them, it does not matter quite so much as when it happens to other people.”* [73, p.312]. Examining implicit assumptions that impact clinical judgment, policies, or process at the point-of-care, whether unintentional or not, is a necessary first step in dismantling untoward harm from barriers created and sustained at the health system level [77]. 

### Micro-level barriers

For people who experience socioeconomic disadvantage, micro-barriers at the level of the individual are interconnected to inequities at the macro-level that can lead to detrimental downstream consequences. Structural inequities such as inadequate housing, poverty, and poor healthcare coverage, racialization, and stigmatization of substance use and mental illness not only result in poor access to healthcare, but also shape how people view accessing healthcare [63]. A number of studies (*n* = 12) illustrate examples of how adverse structural conditions interact with a variety of physical and social factors which further compound barriers in accessing cancer treatment [37, 38, 65, 67, 69, 73, 74, 76, 77–79].

#### Advanced diagnosis and comorbidities

People experiencing socioeconomic disadvantage have higher rates of advanced stage disease at diagnosis as a result of poor access to primary healthcare, cancer prevention strategies and screening [77, 65, 69, 74]. Competing priorities, transportation challenges, medical mistrust, and histories of trauma are among the factors contributing to healthcare ‘avoidance’, leading individuals’ to delay seeking healthcare services until their cancer symptoms become too difficult to manage. [77, 65, 69, 74]. When cancer is diagnosed late, there are often fewer treatment options, and many patients miss the critical time window during which cancer treatment is most effective. As such, three of the studies identified advanced diagnosis as the cause for greater delays in starting treatment because of the need to control progressive cancer symptoms in order to be physically able to withstand treatment. When cancer is diagnosed at an advanced stage, individuals are more prone to hospitalization, leading to interruptions during treatment that hinder further treatment [67, 74, 77]. Similarly,‘comorbidities’ was a key barrier found in nearly one third (n = 6) of the studies [65, 66, 74, 77–79]. Notably, mental health challenges (e.g., depression, anxiety, uncontrolled psychiatric illness) and/or active substance use were identified as significant comorbidities that made adhering to an appointment schedule more difficult, leading to some patients to discontinue their treatment earlier than recommended [65, 74, 77–79]. A few studies acknowledged that individuals with mental health challenges and/or who are actively using substances are often poorly managed and unsupported by the cancer care system; this has been flagged as a key concern among HCPs in the cancer care sector [65, 77]. 

#### Psychosocial dimensions and contexts

Psychosocial dimension of health such as individual values, beliefs, attitudes, socio-cultural and spiritual views are important considerations for healthcare providers in a holistic view of patient-centered care. However, several studies suggested these dimensions were not always considered as essential components of the treatment plan [65, 67, 69, 73, 74]. Failure to include psychosocial and cultural dimensions of care can potentially silo experiences of the treatment journey [73]. Additionally, patient decision-making related to treatment is also influenced by cultural norms, values, and beliefs that are embedded within the broader context of their social environment. A few studies highlighted cancer is considered a ‘death sentence’ for people whose physical and social environments are enmeshed by poverty and systems of oppression [69, 74]. For some, the experience of witnessing their many of peers die from cancer prompted some individuals to distrust the benefit of treatment, and they delayed seeking care [74]. Conversely, for others it swayed their decision towards treatment they believed to be more effective. For example, Bowen and colleagues [69] found women with lower levels of income and breast cancer more frequently chose mastectomy over breast conserving surgery and radiation, as they believed mastectomy was a more *definitive* guarantee that they would not die from their breast cancer [69].

#### Limited social support networks

Limited close social support networks (e.g., friends, family) amplified barriers to treatment for people experiencing socioeconomic disadvantage [65, 76]. In our review, we found this was particularly salient for people who experience structural inequities related to the institutions of marriage [37] and immigration status [65, 76]. For example Levitz et al., [37] found single unmarried women living in poverty and without medical insurance were less likely to receive chemotherapy then their married peers. In this study, structural inequities related to institutions of marriage meant that unmarried women in poverty had less access to diverse sources of income, such as potential medical benefits through their own or spouse’s employer. Likewise, accessing treatment was more challenging for ‘underserved’ Hispanic patients with lung and head and neck cancer who had recently immigrated to the United States who did not have close friends or family [65]. Multiple barriers became more difficult for those who lacked social support networks and did not have assistance with aspects of their care such as communicating with HCPs, transportation, and navigating the cancer care system. As a result, managing side effects and keeping clinical appointments was more challenging, leading to higher rates of nonadherence [65]. Conversely, Costas-Muniz et al. [76], found patients with lower levels of income and who had close social support networks had higher adherence to chemotherapy and/or radiation. In these cases, lower-income was less of a barrier, when close family or friends provided an economic buffer of support such as access to housing, transportation and encouragement to attend appoints. These examples speak to the importance of caregiving relationships in potentially mediating access to cancer treatment.

### Compounding barriers and their intersections

Four studies (20%) reported ‘compounding’ or a ‘multiplicative’ effect for people who face multiple and simultaneous barriers to accessing cancer treatment. That is, for those who experience many barriers in more than one area, were less likely to receive treatment [37, 38, 67, 68], and were more likely to miss treatment visits or experience treatment delays [38, 76]. According to Oduro and colleagues [68], barriers not only acted alone, but interacted and exacerbated the presence of barriers across multiple categories. Notably, timely access from diagnosis to treatment and adherence with a minimal number of missed appointments, are important factors associated with survival among cancer patients. Costas- Muniz et al. [76], found four or more unmet socioeconomic and supportive care needs (i.e., financial support, access to food and nutrition, transportation, housing, social support, health insurance, health legal issues) or those with unstable housing was a significant predictor for a greater number of missed appointments for radiation and/or chemotherapy among a sample of underserved lower-income racialized minorities (i.e., Black and Latino) with cancer. Furthermore, approximately one-third (*n* = 6) of the studies highlighted barriers among people who experience socioeconomic disadvantage and whose experiences of inequitable access to cancer treatment are also shaped by intersecting dimensions of social location such as race/ethnicity, immigration status, Indigenous ancestry, marital status, and gender. For example, Emerson and colleagues [38] highlighted greater disparities in access to breast cancer treatment for Black women in the United States compared with White women of similar age, socioeconomic status (SES), staging and diagnosis, particularly if radiation was involved in their treatment plan [38]. Regardless of Black-White differences, a multivariate analysis of ‘latent class’ (a proxy for the combination of tumor biology, SES, and comorbidities), and presence of a greater number of barriers (e.g., lack of insurance, job loss, financial and transportation issues, comorbidities) resulted in prolonged treatment duration across all treatment modalities (i.e., radiation, chemotherapy), suggesting the experience of facing multiple barriers at once has a compounding effect across the trajectory of cancer treatment [38]. 

## Discussion

In this review, we aimed to map the barriers to accessing cancer treatment among people who experience socioeconomic disadvantage in high-income countries. Among the 20 studies included in our scoping review, we noted a significant proportion (*n* = 16; 80%) of studies originated from the United States with a mixed healthcare system. Comparatively, relatively few studies (*n* = 3; 15%) originated from countries with (primarily) publicly-funded healthcare. Irrespective of geographical and political differences, this review highlighted many universal concepts that showcase inequities in access to cancer treatment for people experiencing socioeconomic disadvantage that arise from the social and structural determinants of health (SSDOH) [81]. We identified that safe and stable housing, transportation, income, and insurance coverage for medical expenses are among the more visible upstream social determinants that impacted access to cancer treatment. Accordingly, these conditions have startling downstream consequences on how cancer care services are utilized across the continuum, and our review underscores their impact on access to treatment. Globally, cancer control organizations have recognized the importance of addressing the SSDOH to close the inequity-gap, however, it is less clear how they should be integrated and operationalized [16]. A commitment from all levels of government to provide funding for healthcare service innovation, universal access to medical care, housing, and social supports, alongside intersectoral collaboration between the health and social care sectors is an initial step forward [27]. 

In addition, a number of studies (*n* = 6) also alluded to how the intersections of social and structural barriers created greater barriers to accessing cancer treatment for racialized minority groups [27, 65, 73, 75, 76, 80]. While addressing implicit bias was brought forward as a recommendation by three articles [73, 77, 78], none explicitly reported the effect of systemic racism as a barrier to accessing cancer treatment. In North America, racialized minorities bear a disproportionate cancer burden, including some of the highest rates of cancer-related morbidity and lowest rates of survival [82, 83]. Such evidence signals how the multifaceted layers of systemic racism perpetuate inequitable access to cancer treatment and exacerbate poor outcomes [82]. For example, a review by Shavers et al. [83]. , found racial/ethnic differences in receipt of definitive primary treatment including adjuvant therapy, surgery, and follow-up after potentially curative treatment, resulted in more frequent disease recurrence and higher cancer-related mortality among Black people in the United States. They also found differences in the receipt of treatment were due to non-clinical factors, and point to differences in referral and prescribing practices. However, the effects of systemic racism and other interlocking forms of structural violence such as colonialism or multiple other forms of discrimination and the impact on access to cancer treatment have not been widely studied. For instance, a scoping review by Horrill and colleagues [17], on barriers to accessing cancer care among Indigenous peoples in Canada found a disproportionate number of studies focused on cancer screening, while only approximately one quarter of the studies focused specifically on diagnosis and treatment. Targeted approaches to understanding the impact of both structural (e.g., political, historical, social factors) and institutional racism (e.g., practices and policies within cancer care organizations) and direct action on dismantling racism in cancer care is required at all levels.

At a meso-level our review underscored how barriers also arise from the way the cancer care systems are organized, designed and delivered. Indeed, half of the included studies (*n* = 10) described barriers to cancer treatment that originated within cancer care organizations, indicating there are factors at the point-of-care that can be modified to improve outcomes [16, 61, 85, 86]. A report issued by the Canadian Medical Association [84] attributes 25% of population health as a direct result of the structure and delivery of the healthcare system. Notably, the World Health Organization also acknowledges that communication failures are the among the leading causes of inadvertent patient harm [87]. Indeed, mismatches in communication between the patient and provider was a focus in almost half of the studies we reviewed (*n* = 9), and either directly or indirectly caused a cascade of difficulties for the patient in understanding complex medical information related to their care, navigating health systems, and safely managing the side effects of treatment. Consequently, some patients experiencing socioeconomic disadvantage became less engaged in their treatment, which led to missed clinical visits, higher rates of nonadherence, or discontinuing treatment earlier than what was recommended [65–71, 78, 80]. As Liu and colleagues [66] emphasize, patient-centered communication has been associated with higher self-efficacy, and has been linked to better adherence to treatment and outcomes. Many of these studies recommended that HCPs take responsibility to address factors that contribute to communication breakdown such as lower health literacy, language barriers, examining bias, and implementing cultural safety practices [65, 67, 71, 73, 77, 78]. These studies also acknowledged the crux of this issue with communication breakdown lay not solely within the dynamics of patient-provider relationships. Rather, health organizations must take responsibility for enabling patient-centered approaches through examining policies and practice standards that may inadvertently disadvantage certain patient population groups. Additionally, organizations have a responsibility to set the tone for inclusivity by shifting potentially harmful workplace norms through providing staff education and training on examining implicit bias, and fostering culturally safe practices [77, 78]. To add, Browne and colleagues [85] have built on these pillars of organizational-level strategies to reduce health inequities at the point-of-care to include trauma- and violence informed care, anti-racism (along with cultural safety), and harm reduction/substance use health. This framework aims to enhance every day healthcare practices at the point-of-care by tailoring approaches to care within the context of peoples’ lives who are at greatest risk for health inequities [85, 86]. 

Likewise, patient-navigation programs have been put forth as a strategy to reduce health inequities, however, our review suggests they are not all created equally, nor are they sufficient as a stand-alone strategy to reduce *all* health inequities within the cancer care system. Relatedly, we found three studies mentioned a lack of continuity between providers [68, 72, 80] The presence of a longitudinal healthcare provider (i.e., social worker, nurse, nurse practitioner, or physician assistant) who was available during the course of the patient’s treatment journey and a well- coordinated cancer care team were also identified as a potential facilitator to breaking down barriers associated with systems fragmentation and mistrust [68, 70]. Inward facing community outreach programs with language specific resources, and promoting the use of peer-support navigators (i.e., lay community workers trained as health educations) was identified as a potential strategy to improve access to cancer treatment for people who are most likely to fall through the cracks of the system [71]. 

Five studies described people who experience socioeconomic disadvantage with severe mental illness and/or substance use are at greater vulnerability for nonadherence to cancer treatment [65, 74, 77–79], however; none of these studies specifically detailed barriers related to this intersection of medicine. Consistent with the available literature, people with cancer who experience severe mental illness (e.g., schizophrenia, bipolar disorder, severe anxiety or depression) and/or substance use have inequitable access to cancer diagnosis and treatment and poor outcomes, which may be the result of stigma [87–91]. For example, Howard et al., describe the process of ‘*diagnostic overshadowing’*, whereby HCPs may minimize or attribute physical complains to psychological or psychiatric causes, and therefore may not fully investigate physical symptoms [88]. This process of overshadowing has led to a range of missed diagnoses, including cancer [88]. Indeed, people who experience severe mental illness with cancer have higher overall mortality rates [87–91]. Additionally, there are specific challenges in treating patients with severe mental illness including higher rates of other comorbidities (cardiovascular disease, obesity, malnourishment, and respiratory disease) that may explain treatment delays for systemic therapy or radiation from post-operative complications and a higher cancer case fatality rate [87–91]. Moreover, some of the cytotoxic drugs used for cancer treatment and anti-nausea medication can have adverse interactions when used in conjunction with certain psychotropic drugs, and may lead to potentially life-threatening side effects [87–91]. Conversely, abrupt discontinuation or switching psychotropic agents to accommodate gold standard approaches to systemic therapy can also lead to a recurrence of severe depression, hallucinations, and withdrawal symptoms [88]. Relatedly, radiation treatment is often given over successive daily fractions, often over several weeks. The safety requirements are such that patients are alone in a treatment room while being given instructions though an automated voice system. Howard et al., describe clinical reports describing this process that can be quite distressing for patients with a tendency for auditory hallucinations, paranoia, or severe anxiety [88]. These examples further speak to the need for integration of mental healthcare with cancer care in treatment planning, cross-disciplinary expertise, and enhanced communication for people who have severe mental illness [77]. However, few studies explore patient experiences with severe mental illness and cancer, and even fewer studies detail how clinical decisions are made for people with severe mental illness or for those who use substances [77]. Research in this area could not only enhance experiences of patient care, but also break down medical silos and stigma for people who face severe mental illness and/or substance use [77].

Lastly, two studies reported on the construct of ‘self-efficacy’ [66, 78], which can be understood as the ability perform certain health behaviors, self-regulation, and motivation to overcome stressful circumstances [92, 93]. Within the cancer treatment sector, self-efficacy has been associated with promoting and maintaining self-care behavior including: ability to cope with cancer and manage side effects, adherence to medication, lifestyle management, and systems navigation [9, 92]. Previous studies have demonstrated that among cancer patients, high self-efficacy was associated with better tolerance and adherence to treatment, less symptom burden, and improved emotional, functional, and social well-being [94, 95]. Higher self-efficacy among patients with cancer has been associated with enhanced communication with HCPs with greater involvement in treatment decision making and satisfaction in their care [94, 95]. However, this paradigm is grounded on many assumptions and social contexts that enable individuals to achieve high self-efficacy. Conversely, our review highlights the multiplicative and competing priorities of daily survival and compounding barriers to accessing cancer treatment. Underlying this tension also rests on the importance of strong social relationships to buffer some of the barriers to cancer treatment related to unmet social care needs. Overarching this phenomenon is the concept of ‘social capital’, that is, the overlapping dimensions of social relationships and access to support and services [96]. Through a social capital lens, we found the strength of relationships to family, friends, community, or HCPs served as a buffer to offset many unmet care needs acting as barriers to receiving or adhering to cancer treatment (i.e., insurance, housing, transportation, emotional support, communication, systems navigation) [76]. However, the focus on social relationships should not be a pre-requisite to receiving care, nor should it absolve social and structural issues that perpetuate poor access to cancer treatment [88]. Along the lines of patient- centered approaches to care, Ayhed and colleagues [96] urge HCPs to use a relational lens to gain insight on how social relationships may leverage access to services or hinder their social mobility. In our review, we found cancer care systems can negate the potential negative effects from a lack of social networks through enhancing care continuity through longitudinal patient-provider relationships, and promoting patient-centered care by examining social contexts [76]. Future research could examine the interplay between self-efficacy and social capital, and how cancer organizations either promote or hinder these dimensions among people or population groups who experience socioeconomic disadvantage.

### Limitations

There may be several limitations to our scoping review. First, our review included high- income countries as defined by the World Bank, however, for feasibility, we limited our search to studies published in the English language, and did not include grey literature, and therefore we may have missed relevant publications.

## Conclusion

Increasingly, there are calls to action on a global scale to redressing cancer-related inequities [5, 16,26,62]. However, the path forward is less clear and requires mobilization at the structural and health systems levels, with particular attention to upstream determinants of health and social processes which create and reinforce multiple and interlocking forms of discrimination (e.g., classism, racism, colonialism, sexism, homophobia, etc. [16, 61, 63]) In the cancer treatment sector, organizations must also examine their structures, policies, and processes at the point-of-care, while simultaneously adopting models of care that are patient-centered and tailored to meet the needs of socioeconomically disadvantaged patients within the context of their social, economic, cultural, and physical environments [61]. Yet as we have highlighted in this review, there is limited evidence to support our understanding of social and structural contexts that both create and sustain barriers in accessing cancer treatment. Further research adopting an intersectional approach and engagement from populations with lived experience of socioeconomic disadvantage may provide more a more nuanced understanding of barriers to cancer treatment, which can then be used to radically shift policy and practice.

### Electronic supplementary material

Below is the link to the electronic supplementary material.

Supplementary Material 1

Supplementary Material 2

## Data Availability

An example of the search strategy has been provided in the supplementary information Inquiries regarding the study protocol and data extraction tool can be directed to AB.

## Abbreviations

HCP: Health care provider
EMR: Electronic medical record
SES: Socioeconomic status
SSDOH: Social and structural determinants of health
