# Supplementary material for: Barriers to cancer treatment for people experiencing socioeconomic disadvantage in high-income countries: a scoping review
Source: BMC Health Serv Res. 2024 May 28;24:670. doi: 10.1186/s12913-024-11129-2 (PMC11134650; doi:10.1186/s12913-024-11129-2)
Supplement: Supplementary file 1 — Supplementary Material 1 [file 12913_2024_11129_MOESM1_ESM.docx]

**Supplementary File 1 Search Methods**

We searched four biomedical databases: (1) (Ovid Medline (R);(2) Ovid Embase; (3) Ovid EBM Reviews-Cochrane Database of Systematic Reviews; and (4) EBSCO CINAHL). We limited the search date to publications from 2008 onwards. This year was chosen in accordance with key/seminal report by the World Health Organization (WHO) n the social determinants of health. To search limit our search to include articles published in high-income countries as defined by the World Bank (2020), a filter was created by an academic librarian (LL) at BC Cancer, adapted from the NICE OECD countries’ geographic search filter for MEDLINE and Embase, published by Ayku and colleagues (2021). No other filters (e.g. language) were applied. The results were imported to Covidence and screened independently by the review team.

**Database search strategies:**

**Ovid MEDLINE(R) and Epub Ahead of Print, In-Process, In-Data-Review & Other Non-Indexed Citations and Daily <1946 to October 28, 2021>**

1.              Homeless Persons/         8705

2.              Working Poor/  17

3.              exp Poverty/      46872

4.              Socioeconomic Factors/ 166462

5.              Economic Factors/           78

6.              Economic Status/             367

7.              Social Class/       42723

8.              ((financially or "socio-economically" or socioeconomically or economically) adj1

(disadvantaged or vulnerable)).mp.              3753

9.              (street adj2 (people or person* or individual* or population* or men or women or man or

woman)).mp. 290

10.           ("lack of housing" or "hard to house" or "substandard housing" or "sub-standard housing" or

"unstably housed" or underhoused or "under housed" or unhoused or squatter* or homeless*

or vagrant* or indigent or "couch surf*" or (sleeping adj3 rough) or "living rough" or "no fixed

abode").mp.              17597

11.           (marginal* adj2 (population* or people* or group* or hous*)).mp.           4302

12.           ("economic insufficiency" or ghetto* or impoverish* or insolven* or "lack of income" or "lack of

money" or ((low or lower or lowest) adj3 (resourced or income* or socioeconomic* or "socio-

economic*" or "social economic*" or financial*)) or "no income" or "no money" or ((poor or

poorer or poorest) adj3 (household* or income* or people or communit* or population? or

socioeconomic or "socio-economic" or "social economic" or financial*)) or poverty or slum or

slums or unemploy* or "low ses").mp.   176272

13.           ((factor* or inequal* or unequal* or equal* or inequit* or equit* or disparit* or gap? or

barrier*) adj3 (income* or socioeconomic* or "socio-economic*" or "social economic* or

economic" or financial*)).mp.                188545

14.           or/1-13 369112

15.           exp Neoplasms/               3561133

16.           Oncology Nursing/           8193

17.           Oncology Service, Hospital/         1514

18.           Cancer Care Facilities/    5750

19.           medical oncology/ or radiation oncology/ or surgical oncology/   25916

20.           (neoplas* or cancer* or tumor* or tumour* or carcinoma* or adenocarcinoma* or sarcoma*

or leiomyosarcoma* or malignan* or oncolog*).mp.             4521605

21.          or/15-20               4872824

22.           exp Drug Therapy/          1433934

23.          exp Therapeutics/           4860352

24.           exp Combined Modality Therapy/             280838

25.           exp Antineoplastic Agents/          1166837

26.           exp Antineoplastic Protocols/     150187

27.           (antineoplastic* or "anti-neoplastic*" or chemotherap* or polychemotherap* or

chemoimmunoradiotherap* or chemoimmunotherap* or chemoradiation or

chemoradiotherap*).mp.             837526

28.           ((anticancer* or "anti-cancer*" or cancer* or cytotoxic*) adj5 (drug* or

agent*)).mp.       152349

29.           ((systemic or hormone or hormonal or endocrine or immune or targeted) adj (therapy or

therapies)).mp.                129330

30.           exp Radiotherapy/          196487

31.           exp Chemoradiotherapy/             17407

32.          Radioimmunotherapy/  3311

33.           Radiotherapy, Adjuvant/              23446

34.           Heavy Ion Radiotherapy/              1137

35.           Radiotherapy, High-Energy/        10261

36.           Radiotherapy, Image-Guided/    3528

37.           Whole-Body Irradiation/               9680

38.           (radiat* or radiother* or irradiat* or radiosurger* or radiochemotherap* or

radioimmunotherap*).mp.                971762

39.           exp Immunotherapy/     298810

40.           Oncolytic Virotherapy/  3326

41.           Immunotherapy, Adoptive/         10918

42.           exp Immune Checkpoint Inhibitors/         10828

43.           exp Antibodies, Monoclonal/      254680

44.           Molecular Targeted Therapy/     32869

45.           (immunotherap* or immunochemotherap* or immunochemoradiotherap*).mp. 138392

46.           (th or su or rt or dt).fs.   6215615

47.           exp Specialties, Surgical/               208817

48.           exp Surgical Procedures, Operative/        3336255

49.           (cryosurg* or transplant* or pneumonectom* or lobectom* or excision* or resection* or

colectomy or hemicolectomy or mastectomy or lumpectomy or laryngectom* or hysterectom*

or surger*).mp.             3596547

50.           or/22-49               11265983

51.           exp Health Services Accessibility/              119506

52.           "Health Services Needs and Demand"/   54596

53.           Quality of Health Care/  75784

54.           Delivery of Health Care/ 101739

55.           exp Delivery of Health Care, Integrated/ 13843

56.           Healthcare Disparities/  20024

57.           "Social Determinants of Health"/              4858

58.           ((health* or care or treatment) adj4 (inequit* or equit* or inequal* or unequal* or equal* or

disparit* or gap? or barrier* or orient* or access* or inaccess*)).mp.         241923

59.           "right to health*".mp.   2490

60.           Medically Underserved Area/     7289

61.           Cost of Illness/  29805

62.           exp "Patient Acceptance of Health Care"/             164906

63.           or/51-62               649845

64.           14 and 21 and 50 and 63               4351

65.           limit 64 to (english language and yr="2008-Current")       3464

66.           65 not ((exp infant/ or exp child/ or adolescent/) not exp adult/) 3292

67.           66 not (exp animals/ not humans.sh.)     3291

68.           67 not (letter not randomized controlled trial).pt.              3262

69.           afghanistan/ or africa/ or africa, northern/ or africa, central/ or africa, eastern/ or "africa, south of the sahara"/ or africa, southern/ or africa, western/ or albania/ or algeria/ or angola/ or argentina/ or armenia/ or azerbaijan/ or bangladesh/ or belize/ or benin/ or bhutan/ or bolivia/ or borneo/ or "bosnia and herzegovina"/ or botswana/ or brazil/ or bulgaria/ or burkina faso/ or burundi/ or cabo verde/ or cambodia/ or cameroon/ or central african republic/ or chad/ or china/ or comoros/ or congo/ or cote d'ivoire/ or cuba/ or "democratic republic of the congo"/ or djibouti/ or dominica/ or dominican republic/ or ecuador/ or egypt/ or el salvador/ or equatorial guinea/ or eritrea/ or eswatini/ or ethiopia/ or fiji/ or gabon/ or gambia/ or "georgia (republic)"/ or ghana/ or grenada/ or guatemala/ or guinea/ or guinea-bissau/ or guyana/ or haiti/ or honduras/ or independent state of samoa/ or exp india/ or indochina/ or indonesia/ or iran/ or iraq/ or jamaica/ or jordan/ or kazakhstan/ or kenya/ or kosovo/ or kyrgyzstan/ or laos/ or lebanon/ or lesotho/ or liberia/ or libya/ or madagascar/ or malaysia/ or malawi/ or mali/ or mauritania/ or mauritius/ or mekong valley/ or melanesia/ or mongolia/ or montenegro/ or morocco/ or mozambique/ or myanmar/ or namibia/ or nepal/ or nicaragua/ or niger/ or nigeria/ or pakistan/ or exp panama/ or papua new guinea/ or paraguay/ or peru/ or philippines/ or "republic of belarus"/ or "republic of north macedonia"/ or romania/ or exp russia/ or rwanda/ or saint lucia/ or "saint vincent and the grenadines"/ or "sao tome and principe"/ or serbia/ or sierra leone/ or senegal/ or somalia/ or south africa/ or south sudan/ or sri lanka/ or sudan/ or suriname/ or syria/ or taiwan/ or tajikistan/ or tanzania/ or thailand/ or tibet/ or timor-leste/ or togo/ or tonga/ or tunisia/ or turkmenistan/ or uganda/ or ukraine/ or uzbekistan/ or vanuatu/ or venezuela/ or vietnam/ or yemen/ or zambia/ or zimbabwe/  1101087

70           andorra/ or "antigua and barbuda"/ or aruba/ or australasia/ or exp australia/ or austria/ or bahamas/ or bahrain/ or baltic states/ or barbados/ or belgium/ or bermuda/ or british virgin islands/ or brunei/ or exp canada/ or exp channel islands/ or chile/ or croatia/ or curacao/ or cyprus/ or czech republic/ or exp denmark/ or exp estonia/ or finland/ or exp france/ or exp germany/ or greece/ or greenland/ or guam/ or hong kong/ or hungary/ or iceland/ or ireland/ or israel/ or exp italy/ or exp japan/ or korea/ or kuwait/ or latvia/ or liechtenstein/ or lithuania/ or luxembourg/ or macau/ or malta/ or micronesia/ or monaco/ or netherlands/ or new caledonia/ or new zealand/ or exp norway/ or oman/ or palau/ or poland/ or polynesia/ or portugal/ or puerto rico/ or qatar/ or "republic of korea"/ or "saint kitts and nevis"/ or san marino/ or saudi arabia/ or exp "scandinavian and nordic countries"/ or seychelles/ or singapore/ or sint maarten/ or slovakia/ or slovenia/ or spain/ or sweden/ or switzerland/ or "trinidad and tobago"/ or united arab emirates/ or exp united kingdom/ or exp united states/ or united states virgin islands/ or uruguay/ or west indies/       3255012

71.           Developed Countries/   20951

72.           70 or 71                3263569

73.           69 not 72             1024122

74.           68 not 73             2826

**EBM Reviews - Cochrane Central Register of Controlled Trials**

1 Homeless Persons/

2 Working Poor/

3 exp Poverty/

4 Socioeconomic Factors/

5 Economic Factors/

6 Economic Status/

7 Social Class/

8 ((financially or "socio-economically" or socioeconomically or economically) adj1 (disadvantaged or vulnerable)).mp.

9 (street adj2 (people or person* or individual* or population* or men or women or man or woman)).mp.

10 ("lack of housing" or "hard to house" or "substandard housing" or "sub-standard housing" or "unstably housed" or underhoused or "under housed" or unhoused or squatter* or homeless* or vagrant* or indigent or "couch surf*" or (sleeping adj3 rough) or "living rough" or "no fixed abode").mp.

11 (marginal* adj2 (population* or people* or group* or hous*)).mp.

12 ("economic insufficiency" or ghetto* or impoverish* or insolven* or "lack of income" or "lack of money" or ((low or lower or lowest) adj3 (resourced or income* or socioeconomic* or "socio-economic*" or "social economic*" or financial*)) or "no income" or "no money" or ((poor or poorer or poorest) adj3 (household* or income* or people or communit* or population? or socioeconomic or "socio-economic" or "social economic" or financial*)) or poverty or slum or slums or unemploy* or "low ses").mp.

13 ((factor* or inequal* or unequal* or equal* or inequit* or equit* or disparit* or gap? or barrier*) adj3 (income* or socioeconomic* or "socio-economic*" or "social economic* or economic" or financial*)).mp.

14 or/1-13

15 exp Neoplasms/

16 Oncology Nursing/

17 Oncology Service, Hospital/

18 Cancer Care Facilities/

19 medical oncology/ or radiation oncology/ or surgical oncology/

20 (neoplas* or cancer* or tumor* or tumour* or carcinoma* or adenocarcinoma* or sarcoma* or leiomyosarcoma* or malignan* or oncolog*).mp.

21 or/15-20

22 exp Drug Therapy/

23 exp Therapeutics/

24 exp Combined Modality Therapy/

25 exp Antineoplastic Agents/

26 exp Antineoplastic Protocols/

27 (antineoplastic* or "anti-neoplastic*" or chemotherap* or polychemotherap* or chemoimmunoradiotherap* or chemoimmunotherap* or chemoradiation or chemoradiotherap*).mp.

28 ((anticancer* or "anti-cancer*" or cancer* or cytotoxic*) adj5 (drug* or agent*)).mp.

29 ((systemic or hormone or hormonal or endocrine or immune or targeted) adj (therapy or therapies)).mp.

30 exp Radiotherapy/

31 exp Chemoradiotherapy/

32 Radioimmunotherapy/

33 Radiotherapy, Adjuvant/

34 Heavy Ion Radiotherapy/

35 Radiotherapy, High-Energy/

36 Radiotherapy, Image-Guided/

37 Whole-Body Irradiation/

38 (radiat* or radiother* or irradiat* or radiosurger* or radiochemotherap* or radioimmunotherap*).mp.

39 exp Immunotherapy/

40 Oncolytic Virotherapy/

41 Immunotherapy, Adoptive/

42 exp Immune Checkpoint Inhibitors/

43 exp Antibodies, Monoclonal/

44 Molecular Targeted Therapy/

45 (immunotherap* or immunochemotherap* or immunochemoradiotherap*).mp.

46 (th or su or rt or dt).fs.

47 exp Specialties, Surgical/

48 exp Surgical Procedures, Operative/

49 (cryosurg* or transplant* or pneumonectom* or lobectom* or excision* or resection* or colectomy or hemicolectomy or mastectomy or lumpectomy or laryngectom* or hysterectom* or surger*).mp.

50 or/22-49

51 exp Health Services Accessibility/

52 "Health Services Needs and Demand"/

53 Quality of Health Care/

54 Delivery of Health Care/

55 exp Delivery of Health Care, Integrated/

56 Healthcare Disparities/

57 "Social Determinants of Health"/

58 ((health* or care or treatment) adj4 (inequit* or equit* or inequal* or unequal* or equal* or disparit* or gap? or barrier* or orient* or access* or inaccess*)).mp.

59 "right to health*".mp.

60 Medically Underserved Area/

61 Cost of Illness/

62 exp "Patient Acceptance of Health Care"/

63 or/51-62

64 14 and 21 and 50 and 63

65 limit 64 to (english language and yr="2008-Current")

66 65 not ((exp infant/ or exp child/ or adolescent/) not exp adult/)

67 66 not (exp animals/ not humans.sh.)

68 67 not (letter not randomized controlled trial).pt.

69 afghanistan/ or africa/ or africa, northern/ or africa, central/ or africa, eastern/ or "africa, south of the sahara"/ or africa, southern/ or africa, western/ or albania/ or algeria/ or angola/ or argentina/ or armenia/ or azerbaijan/ or bangladesh/ or belize/ or benin/ or bhutan/ or bolivia/ or borneo/ or "bosnia and herzegovina"/ or botswana/ or brazil/ or bulgaria/ or burkina faso/ or burundi/ or cabo verde/ or cambodia/ or cameroon/ or central african republic/ or chad/ or china/ or comoros/ or congo/ or cote d'ivoire/ or cuba/ or "democratic republic of the congo"/ or djibouti/ or dominica/ or dominican republic/ or ecuador/ or egypt/ or el salvador/ or equatorial guinea/ or eritrea/ or eswatini/ or ethiopia/ or fiji/ or gabon/ or gambia/ or "georgia (republic)"/ or ghana/ or grenada/ or guatemala/ or guinea/ or guinea-bissau/ or guyana/ or haiti/ or honduras/ or independent state of samoa/ or exp india/ or indochina/ or indonesia/ or iran/ or iraq/ or jamaica/ or jordan/ or kazakhstan/ or kenya/ or kosovo/ or kyrgyzstan/ or laos/ or lebanon/ or lesotho/ or liberia/ or libya/ or madagascar/ or malaysia/ or malawi/ or mali/ or mauritania/ or mauritius/ or mekong valley/ or melanesia/ or mongolia/ or montenegro/ or morocco/ or mozambique/ or myanmar/ or namibia/ or nepal/ or nicaragua/ or niger/ or nigeria/ or pakistan/ or exp panama/ or papua new guinea/ or paraguay/ or peru/ or philippines/ or "republic of belarus"/ or "republic of north macedonia"/ or romania/ or exp russia/ or rwanda/ or saint lucia/ or "saint vincent and the grenadines"/ or "sao tome and principe"/ or serbia/ or sierra leone/ or senegal/ or somalia/ or south africa/ or south sudan/ or sri lanka/ or sudan/ or suriname/ or syria/ or taiwan/ or tajikistan/ or tanzania/ or thailand/ or tibet/ or timor-leste/ or togo/ or tonga/ or tunisia/ or turkmenistan/ or uganda/ or ukraine/ or uzbekistan/ or vanuatu/ or venezuela/ or vietnam/ or yemen/ or zambia/ or zimbabwe/

70 andorra/ or "antigua and barbuda"/ or aruba/ or australasia/ or exp australia/ or austria/ or bahamas/ or bahrain/ or baltic states/ or barbados/ or belgium/ or bermuda/ or british virgin islands/ or brunei/ or exp canada/ or exp channel islands/ or chile/ or croatia/ or curacao/ or cyprus/ or czech republic/ or exp denmark/ or exp estonia/ or finland/ or exp france/ or exp germany/ or greece/ or greenland/ or guam/ or hong kong/ or hungary/ or iceland/ or ireland/ or israel/ or exp italy/ or exp japan/ or korea/ or kuwait/ or latvia/ or liechtenstein/ or lithuania/ or luxembourg/ or macau/ or malta/ or micronesia/ or monaco/ or netherlands/ or new caledonia/ or new zealand/ or exp norway/ or oman/ or palau/ or poland/ or polynesia/ or portugal/ or puerto rico/ or qatar/ or "republic of korea"/ or "saint kitts and nevis"/ or san marino/ or saudi arabia/ or exp "scandinavian and nordic countries"/ or seychelles/ or singapore/ or sint maarten/ or slovakia/ or slovenia/ or spain/ or sweden/ or switzerland/ or "trinidad and tobago"/ or united arab emirates/ or exp united kingdom/ or exp united states/ or united states virgin islands/ or uruguay/ or west indies/

71 Developed Countries/

72 70 or 71

73 69 not 72

74 68 not 73

**Embase**

1 exp Homelessness/

2 homeless person/ or homeless man/ or homeless woman/

3 exp Lowest income group/

4 Poverty/

5 Socioeconomics/

6 Household Economic Status/

7 Economic Status/

8 Social Class/

9 ((financially or "socio-economically" or socioeconomically or economically) adj1 (disadvantaged or vulnerable)).ti,ab,kf.

10 (street adj2 (people or person* or individual* or population* or men or women or man or woman)).ti,ab,kf.

11 ("lack of housing" or "hard to house" or "substandard housing" or "sub-standard housing" or "unstably housed" or underhoused or "under housed" or unhoused or squatter* or homeless* or vagrant* or indigent or "couch surf*" or (sleeping adj3 rough) or "living rough" or "no fixed abode").ti,ab,kf.

12 (marginal* adj2 (population* or people* or group* or hous*)).ti,ab,kf.

13 ("economic insufficieny" or ghetto* or impoverish* or insolven* or "lack of income" or "lack of money" or ((low or lower or lowest) adj3 (resourced or income* or socioeconomic* or "socio-economic*" or "social economic*" or financial*)) or "no income" or "no money" or ((poor or poorer or poorest) adj3 (household* or income* or people or communit* of population? or socioeconomic or "socio-economic" or "social economic" or financial*)) or poverty or slum or slums or unemploy* or "low ses").ti,ab,kf.

14 ((factor* or inequal* or unequal* or equal* or equit* or inequit* or disparit* or gap? or barrier*) adj3 (income* or socioeconomic* or "socio-economic*" or "social economic*" or economic* or financial*)).ti,ab,kf.

15 or/1-14

16 exp Neoplasm/

17 Oncology Nursing/

18 Cancer Center/

19 exp Cancer Patient/

20 exp Oncology/

21 (neoplas* or cancer* or tumor* or tumour* or carcinoma* or adenocarcinoma* or sarcoma* or leiomyosarcoma* or malignan* or oncolog*).ti,ab,kf.

22 or/16-21

23 exp Therapy/

24 exp Cancer Therapy/

25 Chemotherapy/

26 exp Antineoplastic Agent/

27 Antineoplastic Protocol/

28 (antineoplastic* or "anti-neoplastic" or chemotherap* or polychemotherap* or chemoimmunoradiotherap* or chemimmunotherap* or chemoradiation or chemoradiotherap*).ti,ab,kf.

29 ((anticancer* or "anti-cancer*" or cancer or cytotoxic*) adj5 (drug* or agent*)).ti,ab,kf.

30 ((systemic or hormone or hormonal or endocrine or immune or targeted) adj (therapy or therapies)).ti,ab,kf.

31 Radiotherapy/

32 exp Chemoradiotherapy/

33 Radioimmunotherapy/

34 Radiotherapy, Adjuvant/

35 Heavy Ion Radiotherapy/

36 Radiotherapy, High-Energy/

37 Radiotherapy, Image-Guided/

38 Whole-Body Irradiation/

39 (radiat* or radiother* or irradiat* or radiosurger* or radiochemotherap* or radioimmunotherap*).ti,ab,kf.

40 exp Biological Therapy/

41 (immunotherap* or immunochemotherap* or immunochemoradiotherap*).ti,ab,kf.

42 exp Surgery/

43 (cryosurgery or transplant* or pneumonectomy or lobectomy or excision or resection or colectomy or hemicolectomy or mastectomy or lumpectomy or laryngectomy or hysterectomy or surgery).ti,ab,kf.

44 or/23-43

45 Health Equity/

46 exp Health Care Delivery/

47 Health Disparity/

48 "Social Determinants of Health"/

49 Health Care Quality/

50 ((health* or care or treatment) adj4 (inequit* or equit* or inequal* or unequal* or equal* or disparit* or gap? or barrier* or orient* or access* or inaccess*)).ti,ab,kf.

51 "right to health".ti,ab,kf.

52 or/45-51

53 15 and 22 and 44 and 52

54 limit 53 to (english language and yr="2008-Current")

55 54 not (exp juvenile/ not exp adult/)

56 55 not ((exp animal/ or nonhuman/) not exp human/)

57 56 not (letter not randomized controlled trial).pt.

58 afghanistan/ or africa/ or "africa south of the sahara"/ or albania/ or algeria/ or angola/ or argentina/ or armenia/ or exp azerbaijan/ or bangladesh/ or belize/ or benin/ or bhutan/ or bolivia/ or borneo/ or "bosnia and herzegovina"/ or botswana/ or exp brazil/ or bulgaria/ or burkina faso/ or burundi/ or cape verde/ or cambodia/ or cameroon/ or central africa/ or central african republic/ or chad/ or exp china/ or comoros/ or congo/ or cote d'ivoire/ or cook islands/ or cuba/ or "democratic republic congo"/ or djibouti/ or dominica/ or dominican republic/ or ecuador/ or egypt/ or el salvador/ or equatorial guinea/ or eritrea/ or eswatini/ or ethiopia/ or fiji/ or gabon/ or gambia/ or "georgia (republic)"/ or ghana/ or grenada/ or guatemala/ or guinea/ or guinea-bissau/ or guyana/ or haiti/ or honduras/ or exp india/ or indonesia/ or iran/ or exp iraq/ or jamaica/ or jordan/ or kazakhstan/ or kenya/ or kosovo/ or kiribati/ or kyrgyzstan/ or laos/ or lebanon/ or lesotho/ or liberia/ or libyan arab jamahiriya/ or madagascar/ or malaysia/ or malawi/ or mali/ or mauritania/ or mauritius/ or melanesia/ or moldova/ or mongolia/ or "montenegro (republic)"/ or morocco/ or mozambique/ or myanmar/ or namibia/ or nauru/ or nepal/ or nicaragua/ or niger/ or nigeria/ or niue/ or north africa/ or exp pakistan/ or palesine/ or panama/ or papua new guinea/ or paraguay/ or peru/ or philippines/ or "republic of north macedonia"/ or romania/ or exp russian federation/ or rwanda/ or saint lucia/ or "saint vincent and the grenadines"/ or "sao tome and principe"/ or serbia/ or sierra leone/ or senegal/ or somalia/ or south africa/ or south sudan/ or sri lanka/ or sudan/ or suriname/ or syria/ or taiwan/ or tajikistan/ or tanzania/ or thailand/ or tibet/ or timor-leste/ or togo/ or tonga/ or tunisia/ or turkmenistan/ or uganda/ or ukraine/ or uzbekistan/ or vanuatu/ or venezuela/ or vietnam/ or yemen/ or zambia/ or zimbabwe/

59 andorra/ or "antigua and barbuda"/ or aruba/ or australia/ or "australia and new zealand"/ or austria/ or bahamas/ or bahrain/ or baltic states/ or barbados/ or exp belgium/ or bermuda/ or british virgin islands/ or brunei darussalami/ or exp canada/ or exp channel islands/ or chile/ or croatia/ or curacao/ or cyprus/ or czech republic/ or denmark/ or estonia/ or exp finland/ or exp france/ or exp germany/ or greece/ or greenland/ or guam/ or hong kong/ or hungary/ or iceland/ or ireland/ or israel/ or exp italy/ or japan/ or korea/ or kuwait/ or liechtenstein/ or lithuania/ or luxembourg/ or macau/ or malta/ or micronesia/ or monaco/ or netherlands/ or new caledonia/ or new zealand/ or exp norway/ or oman/ or palau/ or poland/ or polynesia/ or exp portugal/ or puerto rico/ or qatar/ or "south korea"/ or "saint kitts and nevis"/ or san marino/ or saudi arabia/ or scandinavia/ or seychelles/ or singapore/ or sint maarten/ or slovakia/ or slovenia/ or spain/ or sweden/ or switzerland/ or "trinidad and tobago"/ or united arab emirates/ or exp united kingdom/ or exp united states/ or united states virgin islands/ or uruguay/ or west indies/

60 Developed Country/

61 59 or 60

62 58 not 61

63 57 not 62

**CINAHL**

S1 (MH "Homeless Persons") OR (MH "Homelessness") OR (MH "Poverty+") OR (MH "Socioeconomic Factors") OR (MH "Economic Factors") OR (MH "Economic Status") OR (MH "Social Class") OR (“working poor”) OR (financially OR "socio-economically" OR socioeconomically OR economically) N0 (disadvantaged OR vulnerable) OR (street N1 (people OR person* OR individual* OR population* OR men OR women OR man OR woman)) OR ("lack of housing" OR "hard to house" OR "substandard housing" OR "sub-standard housing" OR "unstably housed" OR underhoused OR "under housed" OR unhoused OR squatter* OR homeless* OR vagrant* OR indigent OR "couch surf*" OR (sleeping N2 rough) OR "living rough" OR "no fixed abode") OR (marginal* N1 (population* OR people* OR group* OR hous*)) OR ("economic insufficiency" OR ghetto* OR impoverish* OR insolven* OR "lack of income" OR "lack of money" OR ((low OR lower OR lowest) N2 (resourced OR income* OR socioeconomic* OR "socio-economic*" OR "social economic*" OR financial*)) OR "no income" OR "no money" OR ((poor OR poorer OR poorest) N2 (household* OR income* OR people OR communit* OR population# OR socioeconomic OR "socio-economic" OR "social economic" OR financial*)) OR poverty OR slum OR slums OR unemploy* OR "low ses") OR ((factor* OR inequal* OR unequal* OR equal* OR disparit* OR gap# OR barrier*) N2 (income* OR socioeconomic* OR "socio-economic*" OR "social economic* OR economic" OR financial*))

S2 (MH "Neoplasms+") OR (MH "Hamartoma+") OR (MH "Hamartoma Syndrome, Multiple+") OR (MH "Neoplasms by Histologic Type+") OR (MH "Leukemia+") OR (MH "Leukemia, Lymphocytic+") OR (MH "Leukemia, Lymphocytic, Chronic+") OR (MH "Leukemia, Myeloid+") OR (MH "Leukemia, Myeloid, Acute+") OR (MH "Lymphatic Vessel Tumors+") OR (MH "Lymphoma+") OR (MH "Lymphoma, Non-Hodgkin's+") OR (MH "Plasmacytoma+") OR (MH "Lymphoma, B-Cell+") OR (MH "Lymphoma, T-Cell+") OR (MH "Lymphoma, T-Cell, Cutaneous+") OR (MH "Neoplasms, Complex and Mixed+") OR (MH "Wilms' Tumor+") OR (MH "Neoplasms, Connective and Soft Tissue+") OR (MH "Neoplasms, Adipose Tissue+") OR (MH "Neoplasms, Connective Tissue+") OR (MH "Neoplasms, Muscle Tissue+") OR (MH "Sarcoma+") OR (MH "Lipoma+") OR (MH "Liposarcoma+") OR (MH "Giant Cell Tumors+") OR (MH "Mastocytosis+") OR (MH "Myxoma+") OR (MH "Neoplasms, Bone Tissue+") OR (MH "Neoplasms, Fibrous Tissue+") OR (MH "Mastocytosis, Cutaneous+") OR (MH "Osteosarcoma+") OR (MH "Neoplasms, Fibroepithelial+") OR (MH "Myoma+") OR (MH "Myosarcoma+") OR (MH "Neoplasms, Germ Cell and Embryonal+") OR (MH "Germinoma+") OR (MH "Neuroectodermal Tumors+") OR (MH "Teratoma+") OR (MH "Trophoblastic Neoplasms+") OR (MH "Carcinoma, Neuroendocrine+") OR (MH "Glioma+") OR (MH "Neuroectodermal Tumors, Primitive+") OR (MH "Neuroectodermal Tumors, Primitive, Peripheral+") OR (MH "Neuroblastoma+") OR (MH "Neuroendocrine Tumors+") OR (MH "Melanoma+") OR (MH "Neurilemmoma+") OR (MH "Paraganglioma+") OR (MH "Carcinoid Tumor+") OR (MH "Paraganglioma, Extra-Adrenal+") OR (MH "Neuroma, Acoustic+") OR (MH "Gestational Trophoblastic Neoplasms+") OR (MH "Neoplasms, Glandular and Epithelial+") OR (MH "Adenoma+") OR (MH "Neoplasms, Ductal, Lobular, and Medullary+") OR (MH "Neoplasms, Basal Cell+") OR (MH "Neoplasms, Cystic, Mucinous, and Serous+") OR (MH "Adenoma, Islet Cell+") OR (MH "Adenoma, Pituitary+") OR (MH "Adenomatous Polyps+") OR (MH "Mesothelioma+") OR (MH "Carcinoma+") OR (MH "Adenomatous Polyposis Coli+") OR (MH "Adenocarcinoma+") OR (MH "Carcinoma, Basal Cell+") OR (MH "Carcinoma in Situ+") OR (MH "Adenocarcinoma in Situ+") OR (MH "Carcinoma, Ductal+") OR (MH "Carcinoma, Islet Cell+") OR (MH "Cholangiocarcinoma+") OR (MH "Carcinoma, Renal Cell+") OR (MH "Cervical Intraepithelial Neoplasia+") OR (MH "Carcinoma, Squamous Cell+") OR (MH "Neoplasms, Squamous Cell+") OR (MH "Neoplasms, Gonadal Tissue+") OR (MH "Sex Cord-Gonadal Stromal Tumors+") OR (MH "Neoplasms, Nerve Tissue+") OR (MH "Nerve Sheath Tumors+") OR (MH "Neurofibroma+") OR (MH "Neuroma+") OR (MH "Neurofibromatoses+") OR (MH "Neoplasms, Vascular Tissue+") OR (MH "Hemangioma+") OR (MH "Nevi and Melanomas+") OR (MH "Nevus+") OR (MH "Odontogenic Tumors+") OR (MH "Neoplasms by Site+") OR (MH "Abdominal Neoplasms+") OR (MH "Retroperitoneal Neoplasms+") OR (MH "Breast Neoplasms+") OR (MH "Bone Neoplasms+") OR (MH "Skull Neoplasms+") OR (MH "Jaw Neoplasms+") OR (MH "Digestive System Neoplasms+") OR (MH "Biliary Tract Neoplasms+") OR (MH "Gastrointestinal Neoplasms+") OR (MH "Liver Neoplasms+") OR (MH "Pancreatic Neoplasms+") OR (MH "Esophageal Neoplasms+") OR (MH "Intestinal Neoplasms+") OR (MH "Colorectal Neoplasms+") OR (MH "Colonic Neoplasms+") OR (MH "Rectal Neoplasms+") OR (MH "Anus Neoplasms+") OR (MH "Endocrine Gland Neoplasms+") OR (MH "Multiple Endocrine Neoplasia+") OR (MH "Neoplastic Endocrine-Like Syndromes+") OR (MH "Ovarian Neoplasms+") OR (MH "Thymus Neoplasms+") OR (MH "Thyroid Neoplasms+") OR (MH "Pituitary Neoplasms+") OR (MH "Eye Neoplasms+") OR (MH "Retinal Neoplasms+") OR (MH "Head and Neck Neoplasms+") OR (MH "Facial Neoplasms+") OR (MH "Mouth Neoplasms+") OR (MH "Salivary Gland Neoplasms+") OR (MH "Otorhinolaryngologic Neoplasms+") OR (MH "Leukoplakia, Oral+") OR (MH "Nose Neoplasms+") OR (MH "Pharyngeal Neoplasms+") OR (MH "Oropharyngeal Neoplasms+") OR (MH "Nasopharyngeal Neoplasms+") OR (MH "Hematologic Neoplasms+") OR (MH "Nervous System Neoplasms+") OR (MH "Central Nervous System Neoplasms+") OR (MH "Brain Neoplasms+") OR (MH "Meningeal Neoplasms+") OR (MH "Infratentorial Neoplasms+") OR (MH "Supratentorial Neoplasms+") OR (MH "Hypothalamic Neoplasms+") OR (MH "Paraneoplastic Syndromes, Nervous System+") OR (MH "Peripheral Nervous System Neoplasms+") OR (MH "Cranial Nerve Neoplasms+") OR (MH "Skin Neoplasms+") OR (MH "Sebaceous Gland Neoplasms+") OR (MH "Soft Tissue Neoplasms+") OR (MH "Thoracic Neoplasms+") OR (MH "Heart Neoplasms+") OR (MH "Respiratory Tract Neoplasms+") OR (MH "Lung Neoplasms+") OR (MH "Pleural Neoplasms+") OR (MH "Urogenital Neoplasms+") OR (MH "Genital Neoplasms, Male+") OR (MH "Urologic Neoplasms+") OR (MH "Genital Neoplasms, Female+") OR (MH "Uterine Neoplasms+") OR (MH "Cervix Neoplasms+") OR (MH "Prostatic Neoplasms+") OR (MH "Kidney Neoplasms+") OR (MH "Oncologic Nursing") OR (MH "Radiation Oncology Nursing") OR (MH "Oncology Care Units") OR (MH "Cancer Care Facilities") OR (MH "Oncology+") OR (MH "Oncology Surgery+") OR (MH "Oncologic Care") OR (MH "Cancer Patients") OR neoplas* OR cancer* OR tumor* OR tumour* OR carcinoma* OR adenocarcinoma* OR sarcoma* OR leiomyosarcoma* OR malignan* OR oncolog*

S3 (MH "Drug Therapy+") OR (MH "Chemotherapy, Adjuvant+") OR (MH "Chemotherapy, Cancer+") OR (MH "Drug Delivery Systems+") OR (MH "Drug Therapy, Combination+") OR (MH "Fluid Therapy+") OR (MH "Intravenous Therapy+") OR (MH "Parenteral Nutrition+") OR (MH "Hormone Therapy+") OR (MH "Hormone Replacement Therapy+") OR (MH "Medication Errors+") OR (MH "Photochemotherapy+") OR (MH "Polypharmacy+") OR (MH "Prescriptions, Drug+") OR (MH "Self Administration+") OR (MH "Therapeutics+") OR (MH "Airway Management+") OR (MH "Intubation, Intratracheal+") OR (MH "Alternative Therapies+") OR (MH "Alternative Medical Systems+") OR (MH "Bioelectromagnetic Applications+") OR (MH "Electrotherapy+") OR (MH "Phototherapy+") OR (MH "Manual Therapy+") OR (MH "Natural and Biologically Based Therapies+") OR (MH "Diet Therapy+") OR (MH "Pharmacological and Biological Treatments+") OR (MH "Biological Therapy+") OR (MH "Blood Transfusion+") OR (MH "Cytapheresis+") OR (MH "Blood Component Transfusion+") OR (MH "Immunotherapy+") OR (MH "Immune Checkpoint Inhibitors+") OR (MH "Immunization+") OR (MH "Immunosuppression+") OR (MH "Desensitization, Immunologic+") OR (MH "Blood Component Removal+") OR (MH "Bowel and Bladder Management+") OR (MH "Cardiac Pacing, Artificial+") OR (MH "Catheterization+") OR (MH "Angioplasty+") OR (MH "Balloon Dilatation+") OR (MH "Catheterization, Central Venous+") OR (MH "Catheterization, Peripheral+") OR (MH "Angioplasty, Balloon+") OR (MH "Angioplasty, Laser+") OR (MH "Atherectomy+") OR (MH "Heart Catheterization+") OR (MH "Urinary Catheterization+") OR (MH "Chemoprevention+") OR (MH "Combined Modality Therapy+") OR (MH "Chemotherapy, Adjuvant+") OR (MH "Radiotherapy, Adjuvant+") OR (MH "Photochemotherapy+") OR (MH "Dialysis+") OR (MH "Peritoneal Dialysis+") OR (MH "Drainage+") OR (MH "Transportation of Patients+") OR (MH "Resuscitation+") OR (MH "Emergency Treatment+") OR (MH "Resuscitation, Cardiopulmonary+") OR (MH "Advanced Cardiac Life Support+") OR (MH "Enema+") OR (MH "Hemostatic Techniques+") OR (MH "Embolization, Therapeutic+") OR (MH "Hemostasis, Surgical+") OR (MH "Ischemic Preconditioning+") OR (MH "Laser Therapy+") OR (MH "Keratectomy, Laser+") OR (MH "Lithotripsy+") OR (MH "Nutritional Support+") OR (MH "Osteopathy+") OR (MH "Patient Care+") OR (MH "Catheter Care+") OR (MH "Critical Care+") OR (MH "Ear Care+") OR (MH "Emergency Care+") OR (MH "Eye Care+") OR (MH "Intensive Care, Neonatal+") OR (MH "Catheter Care, Urinary+") OR (MH "Catheter Care, Vascular+") OR (MH "Feeding Tube Care+") OR (MH "Home Health Care+") OR (MH "Life Support Care+") OR (MH "Mouth Care+") OR (MH "Oral Hygiene+") OR (MH "Ostomy Care+") OR (MH "Patient Handling+") OR (MH "Perioperative Care+") OR (MH "Intraoperative Care+") OR (MH "Postoperative Care+") OR (MH "Preoperative Care+") OR (MH "Surgical Wound Care+") OR (MH "Residential Care+") OR (MH "Terminal Care+") OR (MH "Skin Care+") OR (MH "Euthanasia+") OR (MH "Wound Care+") OR (MH "Debridement+") OR (MH "Prosthetic Fitting+") OR (MH "Punctures+") OR (MH "Radiotherapy+") OR (MH "Paracentesis+") OR (MH "Chemoradiotherapy+") OR (MH "Radiotherapy, Computer-Assisted+") OR (MH "Radiofrequency Therapy+") OR (MH "Rehabilitation+") OR (MH "Renal Replacement Therapy+") OR (MH "Continuous Renal Replacement Therapy+") OR (MH "Hemofiltration+") OR (MH "Hemodialysis+") OR (MH "Continuous Arteriovenous Hemodialysis+") OR (MH "Continuous Arteriovenous Hemofiltration+") OR (MH "Continuous Venovenous Hemodialysis+") OR (MH "Continuous Venovenous Hemofiltration+") OR (MH "Respiratory Therapy+") OR (MH "Respiration, Artificial+") OR (MH "Ventilation, High Frequency+") OR (MH "Positive Pressure Ventilation+") OR (MH "Oxygen Therapy+") OR (MH "Sorption Detoxification+") OR (MH "Therapy, Computer Assisted+") OR (MH "Treatment Errors+") OR (MH "Medication Errors+") OR (MH "Combined Modality Therapy+") OR (MH "Chemotherapy, Adjuvant+") OR (MH "Photochemotherapy+") OR (MH "Antineoplastic Agents") OR (MH "Angiogenesis Inhibitors+") OR (MH "Antibiotics, Antineoplastic+") OR (MH "Antimetabolites, Antineoplastic+") OR (MH "Antineoplastic Agents, Alkylating+") OR (MH "Antineoplastic Agents, Hormonal+") OR (MH "Anthracyclines+") OR (MH "Daunorubicin+") OR (MH "Doxorubicin+") OR (MH "Folic Acid Antagonists+") OR (MH "Nitrogen Mustard Compounds+") OR (MH "Cyclophosphamide+") OR (MH "Aromatase Inhibitors+") OR (MH "Camptothecin+") OR (MH "Podophyllin+") OR (MH "Trastuzumab+") OR (MH "Immunosuppressive Agents+") OR (MH "Cyclosporins+") OR (MH "Antineoplastics, Immunosuppressives+") OR (MH "Antineoplastic Agents, Combined") OR (antineoplastic* OR "anti-neoplastic*" OR chemotherap* OR polychemotherap* OR chemoimmunoradiotherap* OR chemoimmunotherap* OR chemoradiation OR chemoradiotherap*) OR ((anticancer* OR "anti-cancer*" OR cancer* OR cytotoxic*) N4 (drug* OR agent*)) OR (systemic OR hormone OR hormonal OR endocrine OR immune OR targeted) N0 (therapy OR therapies) OR (MH "Radiotherapy") OR (MH "Radiotherapy, Adjuvant+") OR (MH "Radiotherapy, Computer-Assisted+") OR (MH "Chemoradiotherapy+") OR (MH "Radioimmunotherapy") OR (MH "Radiotherapy, Adjuvant") OR (radiat* OR radiother* OR irradiat* OR radiosurger* OR radiochemotherap* OR radioimmunotherap*) OR (MH "Immunotherapy") OR (MH "Immunization+") OR (MH "Immunosuppression+") OR (MH "Desensitization, Immunologic+") OR "Oncolytic Virotherap*" OR "Oncolytic virus therap*" OR (MH "Immune Checkpoint Inhibitors+") OR (MH "Antibodies, Monoclonal+") OR (MH "Trastuzumab+") OR "molecular targeted therap*" OR "targeted molecular therap*" OR (immunotherap* OR immunochemotherap* OR immunochemoradiotherap*) OR (MW "DT") OR (MW "RT") OR (MW "SU") OR (MW "TH") OR (MH "Specialties, Surgical+") OR (MH "Oncology Surgery+") OR (MH "Surgery, Plastic+") OR (MH "Surgery, Operative+") OR (MH "Ablation Techniques+") OR (MH "Cautery+") OR (MH "Electrocoagulation+") OR (MH "Laser Therapy+") OR (MH "Angioplasty, Laser+") OR (MH "Keratectomy, Laser+") OR (MH "Cerebrospinal Fluid Shunts+") OR (MH "Anastomosis, Surgical+") OR (MH "Gastroenterostomy+") OR (MH "Assisted Circulation+") OR (MH "Biopsy+") OR (MH "Blood Salvage+") OR (MH "Dilatation and Curettage+") OR (MH "Curettage+") OR (MH "Debridement+") OR (MH "Decompression, Surgical+") OR (MH "Drainage+") OR (MH "Device Removal+") OR (MH "Extracorporeal Circulation+") OR (MH "Suction+") OR (MH "Ultrafiltration+") OR (MH "Hemofiltration+") OR (MH "Hemostasis, Surgical+") OR (MH "Intraoperative Care+") OR (MH "Lymph Node Excision+") OR (MH "Mastectomy+") OR (MH "Microsurgery+") OR (MH "Myotomy+") OR (MH "Neurosurgery+") OR (MH "Minimally Invasive Procedures+") OR (MH "Endovascular Procedures+") OR (MH "Denervation+") OR (MH "Craniotomy+") OR (MH "Sympathectomy+") OR (MH "Stereotaxic Techniques+") OR (MH "Orthopedic Surgery+") OR (MH "Amputation+") OR (MH "Ankle Surgery+") OR (MH "Arthroplasty+") OR (MH "Bone Lengthening+") OR (MH "Arthrodesis+") OR (MH "Arthroplasty, Replacement+") OR (MH "Arthroplasty, Replacement, Knee+") OR (MH "Arthroplasty, Replacement, Shoulder+") OR (MH "Cementoplasty+") OR (MH "Vertebroplasty+") OR (MH "Fracture Fixation+") OR (MH "Hip Surgery+") OR (MH "Knee Surgery+") OR (MH "Orthopedic Prosthesis+") OR (MH "Joint Prosthesis+") OR (MH "Ostomy+") OR (MH "Paracentesis+") OR (MH "Enterostomy+") OR (MH "Thoracostomy+") OR (MH "Perioperative Care+") OR (MH "Preoperative Care+") OR (MH "Postoperative Care+") OR (MH "Surgical Wound Care+") OR (MH "Preoperative Period+") OR (MH "Prostheses and Implants+") OR (MH "Internal Fixators+") OR (MH "Larynx, Artificial+") OR (MH "Bone Screws+") OR (MH "Stents+") OR (MH "Repeat Procedures+") OR (MH "Revascularization+") OR (MH "Reoperation+") OR (MH "Myocardial Revascularization+") OR (MH "Coronary Artery Bypass+") OR (MH "Shunts, Surgical+") OR (MH "Surgery, Cardiovascular+") OR (MH "Heart Surgery+") OR (MH "Vascular Surgery+") OR (MH "Reperfusion+") OR (MH "Cardiac Valve Annuloplasty+") OR (MH "Heart Transplantation+") OR (MH "Angioplasty+") OR (MH "Angioplasty, Balloon+") OR (MH "Atherectomy+") OR (MH "Endarterectomy+") OR (MH "Surgery, Computer-Assisted+") OR (MH "Surgery, Digestive System+") OR (MH "Bariatric Surgery+") OR (MH "Colectomy+") OR (MH "Biliary Tract Surgical Procedures+") OR (MH "Cholecystectomy+") OR (MH "Gastrectomy+") OR (MH "Sphincterotomy+") OR (MH "Pancreas Transplantation+") OR (MH "Surgery, Elective+") OR (MH "Surgery, Endocrine+") OR (MH "Eye Surgery+") OR (MH "Surgery, Laparoscopic+") OR (MH "Cataract Extraction+") OR (MH "Filtering Surgery+") OR (MH "Surgery, Ob-Gyn+") OR (MH "Surgery, Oral+") OR (MH "Surgery, Obstetrical+") OR (MH "Surgery, Gynecologic+") OR (MH "Salpingectomy+") OR (MH "Hysterectomy+") OR (MH "Abortion, Induced+") OR (MH "Cesarean Section+") OR (MH "Orthognathic Surgery+") OR (MH "Surgery, Otorhinolaryngologic+") OR (MH "Ear Surgery+") OR (MH "Surgery, Plastic+") OR (MH "Surgery, Podiatric+") OR (MH "Surgery, Prophylactic+") OR (MH "Surgery, Reconstructive+") OR (MH "Surgery, Urogenital+") OR (MH "Surgical Flaps+") OR (MH "Tissue Expansion+") OR (MH "Surgery, Urologic+") OR (MH "Kidney Transplantation+") OR (MH "Surgery, Urologic, Male+") OR (MH "Urinary Diversion+") OR (MH "Nephrectomy+") OR (MH "Prostatectomy+") OR (MH "Surgical Site+") OR (MH "Suture Techniques+") OR (MH "Thoracic Surgery+") OR (MH "Transplantation+") OR (MH "Ultrasonic Surgical Procedures+") OR (MH "Lithotripsy+") OR (MH "Cell Transplantation+") OR (MH "Replantation+") OR (MH "Organ Transplantation+") OR (MH "Tissue and Organ Harvesting+") OR (MH "Tissue Transplantation+") OR (MH "Lung Transplantation+") OR (MH "Bone Marrow Transplantation+") OR (MH "Surgery, Lung+") OR (MH "Surgical Stapling+") OR cryosurg* OR transplant* OR pneumonectom* OR lobectom* OR excision* OR resection* OR colectom* OR hemicolectom* OR mastectom* OR lumpectom* OR laryngectomy* OR hysterectom* OR surger*

S4 (MH "Health Services Accessibility") OR (MH "Right to Health") OR (MH "Universal Health Care") OR (MH "Health Services Needs and Demand+") OR (MH "Quality of Health Care") OR (MH "Health Care Delivery") OR (MH "Health Care Delivery, Integrated") OR (MH "Managed Care Programs+") OR (MH "Healthcare Disparities") OR (MH “Economic Aspects of Illness”) OR (MH "Social Determinants of Health") OR ((health* OR care OR treatment#) N3 (inequit* OR equit* OR inequal* OR unequal* OR equal* OR disparit* OR gap# OR barrier* OR orient* OR access* OR inaccess*)) OR "right to health*" OR (MH "Patient Compliance+") OR (MH "Patient Dropouts") OR (MH "Consumer Participation") OR "Patient Acceptance of Health Care" OR "Patient Acceptance of Healthcare"

S5 S1 AND S2 AND S3 AND S4

S6 S5 NOT ( ( (MH "Child+") OR (MH "Infant+") OR (MH "Adolescence+") ) NOT
( (MH "Adult+") OR (MH "Aged+") OR (MH "Aged, 80 and Over+") ))

S7 S6 NOT (((MH "Animal Population Groups+") OR (MH "Animals+") OR (MH "Birds+") OR (MH "Organisms, Genetically Modified+") OR (MH "Mammals+") OR (MH "Reptiles+") OR (MH "Primates+") OR (MH "Rodents+")) NOT (MH "Human"))

S8 S7 NOT ((PT "Randomized Controlled Trial") OR (PT "Letter"))

S9 (MH "Afghanistan") OR (MH "Africa") OR (MH "Africa, Northern") OR (MH "Africa South of the Sahara") OR (MH "Algeria") OR (MH "Africa, Central") OR (MH "Africa, Eastern") OR (MH "Africa, Western") OR (MH "Africa, Southern") OR (MH "Albania") OR (MH "Angola") OR (MH "Argentina") OR (MH "Armenia") OR (MH "Azerbaijan") OR (MH "Bangladesh") OR (MH "Belize") OR (MH "Benin") OR (MH "Bhutan") OR (MH "Bolivia") OR (MH "Borneo") OR (MH "Bosnia-Herzegovina") OR (MH "Botswana") OR (MH "Brazil") OR (MH "Bulgaria") OR (MH "Burkina Faso") OR (MH "Burundi") OR (MH "Byelarus") OR (MH "Cape Verde") OR (MH "Cambodia") OR (MH "Cameroon") OR (MH "Central African Republic") OR (MH "Chad") OR (MH "China") OR (MH "Congo") OR (MH "Cote d'Ivoire") OR (MH "Cuba") OR (MH "Democratic Republic of the Congo") OR (MH "Djibouti") OR (MH "Dominica") OR (MH "Ecuador") OR (MH "Egypt") OR (MH "El Salvador") OR (MH "Equatorial Guinea") OR (MH "Eritrea") OR (MH "Ethiopia") OR (MH "Gabon") OR (MH "Gambia") OR (MH "Georgia (Republic)") OR (MH "Ghana") OR (MH "Guatemala") OR (MH "Honduras") OR (MH "Guinea") OR (MH "Guinea-Bissau") OR (MH "Guyana") OR (MH "Haiti") OR (MH "Independent State of Samoa") OR (MH "India") OR (MH "Indonesia") OR (MH "Iran") OR (MH "Iraq") OR (MH "Jamaica") OR (MH "Jordan") OR (MH "Kazakhstan") OR (MH "Kyrgyzstan") OR (MH "Kenya") OR (MH "Laos") OR (MH "Lebanon") OR (MH "Lesotho") OR (MH "Liberia") OR (MH "Libya") OR (MH "Madagascar") OR (MH "Malaysia") OR (MH "Malawi") OR (MH "Mali") OR (MH "Mauritania") OR (MH "Melanesia") OR (MH "Mongolia") OR (MH "Morocco") OR (MH "Mozambique") OR (MH "Myanmar") OR (MH "Namibia") OR (MH "Nepal") OR (MH "Nicaragua") OR (MH "Niger") OR (MH "Nigeria") OR (MH "Pakistan") OR (MH "Panama+") OR (MH "Papua New Guinea") OR (MH "Paraguay") OR (MH "Peru") OR (MH "Philippines") OR (MH "Macedonia (Republic)") OR (MH "Romania") OR (MH "Russia") OR (MH "Rwanda") OR (MH "Serbia") OR (MH "Sierra Leone") OR (MH "Senegal") OR (MH "Somalia") OR (MH "South Africa") OR (MH "Sudan") OR (MH "Suriname") OR (MH "Sri Lanka") OR (MH "Taiwan") OR (MH "Tajikistan") OR (MH "Tanzania") OR (MH "Thailand") OR (MH "Tibet") OR (MH "East Timor") OR (MH "Togo") OR (MH "Tunisia") OR (MH "Turkmenistan") OR (MH "Uzbekistan") OR (MH "Uganda") OR (MH "Ukraine") OR (MH "Venezuela") OR (MH "Vietnam") OR (MH "Yemen") OR (MH "Zambia") OR (MH "Zimbabwe") OR comoros OR eswatini OR fiji OR grenada OR indochina OR kosovo OR mauritius OR "mekong valley" OR montenegro OR "saint lucia" OR "saint vincent and the grenadines" OR "sao tome and principe" OR tonga OR Vanuatu

S10 (MH "Andorra") OR (MH "Antigua") OR (MH "Australia+") OR (MH "New Zealand") OR (MH "Austria") OR (MH "Bahamas") OR (MH "Bahrain") OR (MH "Baltic States") OR (MH "Barbados") OR (MH "Belgium") OR (MH "Brunei") OR (MH "Canada+") OR (MH "Chile") OR (MH "Croatia") OR (MH "Czech Republic") OR (MH "Denmark") OR (MH "Estonia") OR (MH "Finland") OR (MH "France") OR (MH "Germany+") OR (MH "Greece") OR (MH "Greenland") OR (MH "Guam") OR (MH "Hong Kong") OR (MH "Hungary") OR (MH "Iceland") OR (MH "Ireland") OR (MH "Italy") OR (MH "Israel") OR (MH "Japan") OR (MH "South Korea") OR (MH "Kuwait") OR (MH "Liechtenstein") OR (MH "Lithuania") OR (MH "Luxembourg") OR (MH "Macao") OR (MH "Micronesia") OR (MH "Monaco") OR (MH "Netherlands") OR (MH "Norway") OR (MH "Oman") OR (MH "Poland") OR (MH "Polynesia") OR (MH "Portugal") OR (MH "Puerto Rico") OR (MH "Qatar") OR (MH "San Marino") OR (MH "Saudi Arabia") OR (MH "Scandinavia") OR (MH "Sweden") OR (MH "Slovakia") OR (MH "Spain") OR (MH "Switzerland") OR (MH "United Kingdom+") OR (MH "Great Britain+") OR (MH "Trinidad and Tobago") OR (MH "United Arab Emirates") OR (MH "United States+") OR (MH "United States by Individual State+") OR (MH "Virgin Islands of the United States") OR (MH "Uruguay") OR (MH "West Indies") OR Aruba OR Australasia OR "British Virgin Islands" OR "Channel Islands" OR Curacao OR Cyprus OR Malta OR "New Caledonia" OR Palau OR "Saint Kitts and Nevis" OR Seychelles OR (MH "Developed Countries")

S11 S9 NOT S10

S12 S8 NOT S11

S13 S8 NOT S11 **Limiters** - Published Date: 20080101-

S14 S8 NOT S11

**EBM Reviews - Cochrane Database of Systematic Reviews**

1 ((financially or "socio-economically" or socioeconomically or economically) adj1 (disadvantaged or vulnerable)).ti,ab,kw.

2 (street adj2 (people or person* or individual* or population* or men or women or man or woman)).ti,ab,kw.

3 ("lack of housing" or "hard to house" or "substandard housing" or "sub-standard housing" or "unstably housed" or underhoused or "under housed" or unhoused or squatter* or homeless* or vagrant* or indigent or "couch surf*" or (sleeping adj3 rough) or "living rough" or "no fixed abode").ti,ab,kw.

4 (marginal* adj2 (population* or people* or group* or hous*)).ti,ab,kw.

5 ("economic insufficiency" or ghetto* or impoverish* or insolven* or "lack of income" or "lack of money" or ((low or lower or lowest) adj3 (resourced or income* or socioeconomic* or "socio-economic*" or "social economic*" or financial*)) or "no income" or "no money" or ((poor or poorer or poorest) adj3 (household* or income* or people or communit* or population? or socioeconomic or "socio-economic" or "social economic" or financial*)) or poverty or slum or slums or unemploy* or "low ses").ti,ab,kw.

6 ((factor* or inequal* or unequal* or equal* or inequit* or equit* or disparit* or gap? or barrier*) adj3 (income* or socioeconomic* or "socio-economic*" or "social economic* or economic" or financial*)).ti,ab,kw.

7 or/1-6

8 (neoplas* or cancer* or tumor* or tumour* or carcinoma* or adenocarcinoma* or sarcoma* or leiomyosarcoma* or malignan* or oncolog*).mp.

9 (antineoplastic* or "anti-neoplastic*" or chemotherap* or polychemotherap* or chemoimmunoradiotherap* or chemoimmunotherap* or chemoradiation or chemoradiotherap*).mp.

10 ((anticancer* or "anti-cancer*" or cancer* or cytotoxic*) adj5 (drug* or agent*)).mp.

11 ((systemic or hormone or hormonal or endocrine or immune or targeted) adj (therapy or therapies)).mp.

12 (radiat* or radiother* or irradiat* or radiosurger* or radiochemotherap* or radioimmunotherap*).mp.

13 (immunotherap* or immunochemotherap* or immunochemoradiotherap*).mp.

14 (cryosurg* or transplant* or pneumonectom* or lobectom* or excision* or resection* or colectomy or hemicolectomy or mastectomy or lumpectomy or laryngectom* or hysterectom* or surger*).mp.

15 or/9-14

16 ((health* or care or treatment) adj4 (inequit* or equit* or inequal* or unequal* or equal* or disparit* or gap? or barrier* or orient* or access* or inaccess*)).ti,ab,kw.

17 "right to health*".ti,ab,kw.

18 or/16-17

19 7 and 8 and 15 and 18

20 limit 19 to last 13 years
