# Supplementary material for: Barriers to cancer treatment for people experiencing socioeconomic disadvantage in high-income countries: a scoping review
Source: BMC Health Serv Res. 2024 May 28;24:670. doi: 10.1186/s12913-024-11129-2 (PMC11134650; doi:10.1186/s12913-024-11129-2)
Supplement: Supplementary file 2 — Supplementary Material 2 [file 12913_2024_11129_MOESM2_ESM.docx]

**Supplementary File 2.** **Summary of Included Articles**

| **Author(s)/**  **year** | **County of**  **origin/ setting** | **Study**  **methodology** | **Description of**  **population** | **Aims** | **Examples of barriers identified:** |
| --- | --- | --- | --- | --- | --- |
| Borraya et al.  2020 | United States/  Safety net hospital | Qualitative:  Ethnography | Underserved (e.g., low income, uninsured) Hispanic cancer patients with head and neck cancer, their caregivers and providers | Explore treatment related challenges and support needs | - Low health literacy, language barriers and poor communication from healthcare providers (HCPs) affects patients’ ability to adhere to treatment. Most patients did not know the diagnosis, stage of their cancer or reasons they underwent certain treatment procedures - Costs associated with transportation and insurance co-payments, job loss. - Lack of housing or poor living conditions - ‘Undocumented’ recent immigrants have more difficulty accessing health care, and getting the drugs they need for cancer treatment. |
| Bowen et al.  2013 | United States/  State funded public assistance program | Qualitative:  Grounded theory | Low-income women with breast cancer | Investigates what occurs after low- income women receive an abnormal screening and the factors that influence their decisions and behavior | - Decisions about the course of their treatment were determined by experiential knowledge gained by their environment. For example, some women opted to have an unnecessary mastectomy versus lumpectomy due to fear of breast cancer related death. - Structural barriers conceptualized as lack of transportation and rural geographic status - Poor provider communication, inaccessible medical language, and patterns of unequal power relations contributed to patterns of silence and frustration impeded adherence to treatment and follow- up appointments. - Lack of insurance or suboptimal insurance led to choosing suboptimal treatment because of cost. |
| Byrne et al.  2018 | Ireland/  Oncology unit at regional catchment hospital | Qualitative:  Thematic analysis outlined by Braun and Clarke (2006) | Low-income women with breast cancer | Explore potential barriers to cancer care and uncover themes related to disadvantage and survival | - Unstable housing and threats of eviction during treatment - Inadequate insurance led to difficulty paying for direct aspects of healthcare - Financial problems interfering with receiving healthcare (e.g., not being able to pay for food bills) - Transportation and distance from healthcare facility - Not having childcare - Employment demands makes getting healthcare more difficult - Lower health literacy and poor communication with HCPs: information not provided or explained. - Delays in diagnosis and accessing diagnostic and ancillary services: more than half the women were delayed from one to 24 months in seeking medical attention. - ‘System problems’- long wait times, unable to reach HCP by telephone, opening hours are not convenient. |
| Costas-Muniz  et al.  2016 | United States/  Cancer treatment facility | Quantitative:  Self- reported survey | Low-income Latino and Black cancer patients | Determine if unmet financial, logistic and supportive care needs predict self-reported adherence to cancer treatment appointments of chemotherapy and/or radiation | - After adjusting for demographic and health related factors, patients needing supportive care services were more than two times as likely to report missed appointments for their cancer treatment. In the adjusted analyses, patients needing assistance with health insurance, health law, housing issues, and with more than four unmet needs were more than three times as likely to report missed appointments and are at risk for being non-adherent to cancer treatment, regardless of their ethnicity and/or race (pp. 6-7). - Patients who face housing instability are at especially risk for not adhering to chemotherapy and/ or radiation. - Competing priorities of daily survival may cause patients to deprioritize their medical needs. |
| Crawford et al.  2009 | United Kingdom/  National cancer registry data | Quantitative:  Retrospective cohort | Patients with lung cancer facing deprivation and geographical challenges | Study the influence of deprivation and geographic access to specialist services and their influence on treatment and access to health services | - Attainment of histological diagnosis and receipt of treatment (surgery, radiotherapy, or chemotherapy) decreased for people who faced worse socioeconomic deprivation and who lived further away from a cancer treatment center. |
| Darby et al.  2009 | United States/  Multiple | Qualitative:  Grounded Theory | African American medically underserved (e.g., no insurance, Medicaid, or Medicare) women with breast cancer | Explore the financial burden of breast cancer on African American medically underserved women | - Inadequate insurance-“the type of insurance seems to dictate the type of treatment you get..” (p. 724) - Unemployment and financial difficulties to cover cost of out-of-pocket expenses (i.e., childcare, transportation) resulted in missed treatment visits. |
| Emerson et al.  2020 | United States/  State cancer registry data | Quantitative:  Population-based cohort study | Latent classes (i.e., socioeconomic status, comorbidity factors, access to care factors, and tumor characteristics)  Black and White women with stage I-III breast cancer | Assess the role of tumor biology and access factors in both treatment delay, and treatment duration using LCA (latent class analysis) | - Compounding barriers: individuals who experienced one form of delay were more likely to experience additional types of delays - Prolonged treatment duration was associated with uninsured, financial, and transportation issues. - Black women with low SES and more barriers to care had substantial proportions (32.9% and 42.6%; respectively) of prolonged treatment duration, especially when radiation therapy was part of treatment. |
| Facer et al.  2021 | United States/  Multiple:  Non-government (state) homeless health services program | Quantitative:  Prospective cohort study | Persons experiencing homelessness (PES) with cancer | Assess adherence to radiation therapy treatment plans in PEH with cancer at a large academic medical center | - Most common documented reasons for missed visits, included: transportation challenges (25%), drug overdose (8%) and hospitalization (7%) (p. 1022). - Three patients with significant psychiatric co- comorbidities (e.g., schizophrenia, prior suicidal ideation) and substance use disorders (e.g., alcohol, opioids) had the highest rates of non-adherence to radiation. - Many in this population often do not have access to conventional means of communication (e.g., cell phone, email) which presents an additional barrier to care coordination. |
| Festa et al.,  2020 | United States/  Safety net hospital | Mixed methods:  Small scale descriptive study | Homeless women with breast cancer | Assess length of delay and barriers to breast cancer treatment among women experiencing homelessness as it related to type of homelessness and assess reasons for treatment delay | - Those who were chronically homeless experienced significantly more delays to first treatment than those who were episodically or transitionally homeless (p. 453). - Lacking a recovery shelter post breast cancer surgery - Majority of the study population became homeless after receiving their diagnosis or directed after their first treatment, and this led to delays in receiving treatment (p. 454). - Women with the longest delays were also those who were the most transient (moving between shelters frequently) at the time of their diagnosis and treatment (p. 454). - Several women missed treatment appointments due to documented psychiatric issues, comorbidities, or substance use (p. 454). - Financial concerns related to medical expenses. |
| Gould et al.,  2009 | Canada/  Not specified | Qualitative:  Not specified | Women from multiple social locations (i.e., lower income, older age, Indigenous identity) with breast cancer or gynecological cancer | Explore experiences of older, lower-income, and ‘Aboriginal’ women diagnosed with cancer and treated by the cancer system in Ontario, Canada. | - Several lower-income women who were uninsured, or were unaware or ineligible for, public insurance programs also found it a challenge to afford supportive care drugs such as anti-nausea pills - A few women decided against receiving treatment because of the way the health and social care systems are organized. - In some cases, women perceived discrimination from medical professionals, in others women's social locations and circumstances were not taken into account or accommodated by the health system. - Many women in this study did not know about services or did not have accurate information about cancer itself. |
| Jerome D’Emilia et al.  2021 | United States/  State funded public assistance program | Qualitative:  Descriptive | Low-income women with breast cancer enrolled in the National Breast and Cervical Early Detection Program | Determine if there were delays in diagnosis and treatment and the cause of these delays. | - Delays to start of treatment due to poor quality of care in receiving a definitive diagnosis, and lack of access to preventive care services due to systems fragmentation. - Lower health literacy and poor provider communication: lack of understanding the disease process such as type of cancer, stage at diagnosis and inconsistency in reporting may be related to a lack of understanding of the disease process, which could impede the pursuit of care (p. e47). - Incomplete treatment due to financial toxicity |
| Lawrie et al.  2020 | Multiple: United States, Canada, Australia, Northern Ireland | Scoping review | People experiencing homelessness with cancer | Examine and summarize what is known about homelessness, cancer, and health literacy | - Lower health literacy affects patient's ability to access and utilize the health care system - If system navigation is too difficult, then actions such as cancer screening, preventive care, proper follow up and keeping clinical appointments will be seen as less important than competing social conditions (e.g. being mobile) and life priorities (e.g., finding housing), and therefore not acted upon. (p. 93) - Two articles referenced barriers to treatment adherence include: cost, difficulty storing or refrigerating medications, theft of belongings, and mental health or addiction problems" p. 94 - "Compared with having greater health literacy, patients with low health literacy may have less comfort with medical interactions, less knowledge about the importance of accessing health care, concerns about how low literacy knowledge being exposed, and feelings of shame” (p. 94) - Past negative encounters with the healthcare system, feelings of discrimination and stigmatization whether actual or perceived make the homeless reluctant to access and utilize health care, pessimism about treatment, and mistrust. - Lack of disease knowledge influences how patients interact with health care providers. Patients with low health literacy may be more passive in decision making around their health and reluctant to ask questions of the health care providers (p. 95). - Many healthcare providers are unaware of their patient's low literacy levels, but even healthcare providers with this knowledge may be unable to communicate successfully and build the literacy of their patients. - Poor provider communication: health care providers may lack the skills necessary to communicate clearly and evaluate a patient's comprehension, may lack engagement, or may face time pressures that make for a "fast-paced and potentially anxiety-driven encounters". Patients are deeply reluctant to ask questions and participate in health care decision making (p. 95). - Health care provider bias can be noted in a reluctance to serve a population who cannot comply with self-care regimens or medication adherence. Homeless patients are often labeled as noncompliant by medical staff when the social stressors of homelessness like lack of transportation, cause them to miss treatment appointments (p. 95) |
| Leal et al.  2018 | United States/  Safety net hospital | Qualitative:  Focused ethnography | Disadvantaged patients (i.e., low-income, primarily racialized minorities, no insurance) with colorectal cancer at a Safety Net Hospital | To understand and explore vulnerable patients’ perceptions and experiences of factors impacting care for colorectal cancer | - Limited resources for accessing care including: economic constraints, no medical insurance, low health literacy, and no language specific information - "many stated the economic constraints required them to choose between caring for their health and supporting themselves financially” (p. 507). |
| Levitz et al.  2015 | United States/  State cancer registry data | Quantitative:  Retrospective cohort study | Unmarried and inadequately insured woman living in poverty with colon cancer | Examines multiplicative disadvantage of being an unmarried and inadequately insured woman living in poverty on receipt of chemotherapy | - Uninsured or underinsured living in poverty unmarried women are less likely than married women with non-localized colon cancer to receive indicated chemotherapy. - People who living in poverty may have been less able to pick up on the co-insurance costs and co-payments - Unmarried women, living in poverty may have less diverse sources of income. |
| Lineback et al.  2017 | United States/  Multiple | Qualitative:  Multiple methods (i.e., semi-structured interviews and online surveys) | Patients and family members of patients with the diagnosis of esophageal cancer from both high and low SES populations. | To provide an understanding of barriers to optimal esophageal cancer care, and to understand how to improve outcomes for patients with low SES. | - Patients with lower socioeconomic (SES) circumstances did not have a good understanding of their treatment options compared to those with higher-SES reported greater comprehension of their treatment options (p. 418). - Patients with lower-SES were significantly less likely to seek out second opinions from oncologists or surgeons after the initial diagnosis. - More patients from the low-SES group reported losing trust in at least one of their physicians over the course of their diagnosis and treatment (50%) when compared to high-SES group (15.4%) (p. 418). - Patients with lower- SES group lost their employment (33.3%) in low-SES group than high-SES group (2.6%). Loss of employment and loss of insurance was reported as a major influence in choosing care centers or treatment options (p. 418-419). - A number of patients with low-SES noted that when provided with a care team manager or social worker, hospital systems were often less able to solve these issues for them (p. 419). - A significantly smaller portion of patients from the low-SES group were offered surgical treatment, either alone or with neo-adjuvant chemo-radiation (44.7%), than the high- SES group (76.3%) (p. 418). |
| Liu et al.  2013 | United States/  State survey | Quantitative:  Survey data | Low-income and medically underserved women with breast cancer | To assess the impact of patient-provider communication on adherence to tamoxifen and aromatase inhibitors 36 months after breast cancer. | - Side effects from hormone treatment had a negative impact on adherence (p. 3). - Not having health insurance was a major barrier to hormone treatment adherence (p.4). - Patients with less self- efficacy in patient- physician interactions were less likely to adhere to hormone therapy (p.5). |
| Noel et al.  2015 | United States/  Multiple | Mixed methods | Low-SES African American women with breast cancer, and health work force participants (i.e., patient navigators, clinic providers) | Examines provider-level factors influencing the inability to complete treatment | - African American women who did not receive surgery as a first course of treatment tended to be older, have public insurance, less likely to have localized tumors, and had tumors that were larger (p.8). - Limited knowledge of breast cancer treatment and purpose of treatment - Difficulty processing treatment information due to nerves and the speed at which it was presented to them (p.8). - Radiation was the least understood by women in the study in terms of connection to survival. Perceived as optional rather than linking it with survival (p.10). - Decision making was not shared (p.10). - Communication breakdown between patient-provider in comparing EMR entries with patient narratives, we noted a disconnection between what women described as recommended or optional treatments and what physicians recorded in the EMR (p. 10). - Failure of continuity of service- navigator service tapers off after surgery, with only one employed by medical oncology and none by radiation oncology (p.10). - Fragmentation of services- led to increased delays in treatment initiation (p.8). - Navigators reported receiving women's contact information by a primary care physician after the women received a confirmed or probable diagnosis, yet in many cases, by the time they called the contact information was no longer valid (p.10). - Navigators reported frustration in trying to deal with patient emotions by phone. This was particularly acute in cases where the women were frightened at the beginning of treatment or on the verge of dropping out. (p.8) |
| Oduro et al.  2013 | United States/  State funded public assistance program | Qualitative:  Grounded theory | Low-income Latino men with prostate cancer | To identify barriers to (cancer) care from the perspective of Latino men with prostate cancer | - Lower-SES together with limited or no medical coverage- forced participants to choose between costly out of pocket payments or delaying care (p. 378). - Financial burden of complying with follow up recommendations (after screening) functioned as a barrier to accessing health care leading to delays in their diagnosis and/ or treatment (p.378). - Competing priorities of daily living. Inability to work during treatment threatened to exacerbate their economic situation (p.378). - System barriers: numerous hospital visits, multiple and changing providers, difficulty in scheduling doctors' appointments, extended office wait times, and malfunctioning equipment heightened feelings of anxiety, distrust, and the belief that they were receiving inferior care - Inadequate communication coupled with poor continuity intensified feelings of mistrust of physicians and the medical system, complicating participants' treatment (p.378) - Lower health literacy and language barriers contributed to misunderstandings of treatment options and doctors' recommendations hindered participant access to adequate care and information (p.378) - Miscommunication regarding the treatment options and their treatment plan left participants disempowered. Many participants did not understand their treatment options, what would happen during treatment, and the potential side effects of treatment. This resulted in treatment regret (p. 378) - Compounding effect when barriers exist simultaneously (p. 379) |
| Smith et al.  2014 | United States/  Cancer treatment facility | Qualitative:  Descriptive | Health care professionals: social workers and financial counselors working with patients with cancer. | Describe financial assistance resources available to social workers and financial care counselors at a referral cancer center and exploration of barriers to financial services | - Limitations of available financial resources and eligibility criteria that are constantly changing. - Change of income status into poverty during treatment: "We feel so helpless as there are no resources to work with. Patients are losing their homes to pay for treatment and are forced to living in their automobiles” (p. e369). - Patients can't afford oral their oral medications so they cut them in half - Lack of temporary and semi-permanent lodging (p. e 370) - Patients may be too embarrassed to ask for financial resources (p. e370) - Institutional infrastructure as a barrier: according to one social worker, "our clinic is a barrier as well- patient's don't know how to find us (p. e370) - Patients unaware of resources available to them - Time constraints: not enough time to address all patient's cancer related needs in one appointment (p. e 370). - Resource limitations (e.g., stringent eligibility criteria, decreasing funds), barriers to access (e.g., limited social workers to identify and refer for services, patient reluctance to discuss finance distress), and process inefficiencies often lead to unmet needs (p. e 371) |
